# Dendritic compartment-specific spine formation in layer 5 neurons underlies cortical circuit maturation during adolescence

**DOI:** 10.1126/sciadv.adw8458

**Published:** 2026-01-14

**Authors:** Ryo Egashira, Meng-Tsen Ke, Nao Nakagawa-Tamagawa, Satoshi Fujimoto, Shigenori Inagaki, Tsuyoshi Takagi, Tsuyoshi Miyakawa, Yoshiaki Tagawa, Takeshi Imai

**Affiliations:** ^1^ Graduate School of Medical Sciences, Kyushu University, Fukuoka 812-8582, Japan.; ^2^ RIKEN Center for Developmental Biology, Kobe 650-0047, Japan.; ^3^ Department of Physiology, Graduate School of Medical and Dental Sciences, Kagoshima University, Kagoshima 890-8544, Japan.; ^4^ Institute for Developmental Research, Aichi Developmental Disability Center, Kasugai, Aichi 480-0392, Japan.; ^5^ Division of Systems Medical Science, Center for Medical Science, Fujita Health University, Toyoake, Aichi 470-1192, Japan.

## Abstract

The development of cognitive functions continues into adolescence. However, it is not fully understood how cortical circuitry changes during adolescence. Here, we performed a comprehensive super-resolution mapping of dendritic spines in layer 5 extratelencepharic-projecting (L5 ET) neurons in the primary somatosensory cortex in mice. In adults, the dendritic spines are highly enriched in the middle compartment of the apical dendrites (spine density “hotspot”), where dendritic calcium spikes are generated. In early development, dendritic spines are evenly distributed. During adolescence, however, the spine density increases specifically in the middle compartment of the apical dendrites in an experience-dependent manner, while other dendritic compartments show a slight reduction. Furthermore, spine accumulation at the hotspot was specifically impaired in mouse models of schizophrenia, demonstrating a link between adolescent spine formation and neuropsychiatric disorders. Our finding suggests that the dendritic compartment-specific spine formation during adolescence shapes nonlinear dendritic integration in L5 ET neurons and supports the maturation of cognitive functions.

## Teaser

Mouse models of schizophrenia show defects in adolescent spine formation in a specific dendritic compartment of L5 neurons.

## INTRODUCTION

Neurons in the brain communicate with each other through synapses. A typical pyramidal neuron in the brain receives synaptic inputs from ~10^4^ synapses (*1*). Many of the excitatory synapses are formed at a small protrusion of dendrites, known as dendritic spines (*2*), while inhibitory ones are formed at spines, dendritic shafts, and somata (*3*, *4*). It has been known that the density of dendritic spines dynamically changes in the neocortex during postnatal development. It is generally believed that the density of spines increases during early postnatal stages (childhood) and then declines during adolescence to form mature cortical circuit (*5*–*7*). It is also considered that the dysregulation of spine density (i.e., formation and elimination) is associated with various neuropsychiatric diseases (*8*, *9*). For example, it has been proposed that excessive spine “pruning” is associated with schizophrenia (*10*). However, earlier studies are mostly based on the average synapse density or quantification of just small dendritic segments due to technical limitations. Therefore, it is yet to be established whether and how spine density is controlled at the whole-neuron scale during the cortical circuit maturation during adolescence.

Accumulating evidence suggests the importance of spatial distribution of dendritic spines for dendritic integration. Synaptic inputs at different locations have different impacts on the neuron. For example, nearby spines are often coactivated, known as synapse clustering (*11*–*15*). Different branches of a dendrite tend to differentiate their inputs to control distinct functions (*16*–*18*). Distal and proximal parts of dendrites also have distinct impacts for synaptic integrations (*19*–*22*). Some of these parameters are also important to generate dendritic spikes, important for supralinear and compartmentalized integration of synaptic inputs (*23*–*25*). Thus, the location of dendritic spines has to be tightly controlled for efficient synaptic integration and plasticity (*26*).

**Fig. 1.**
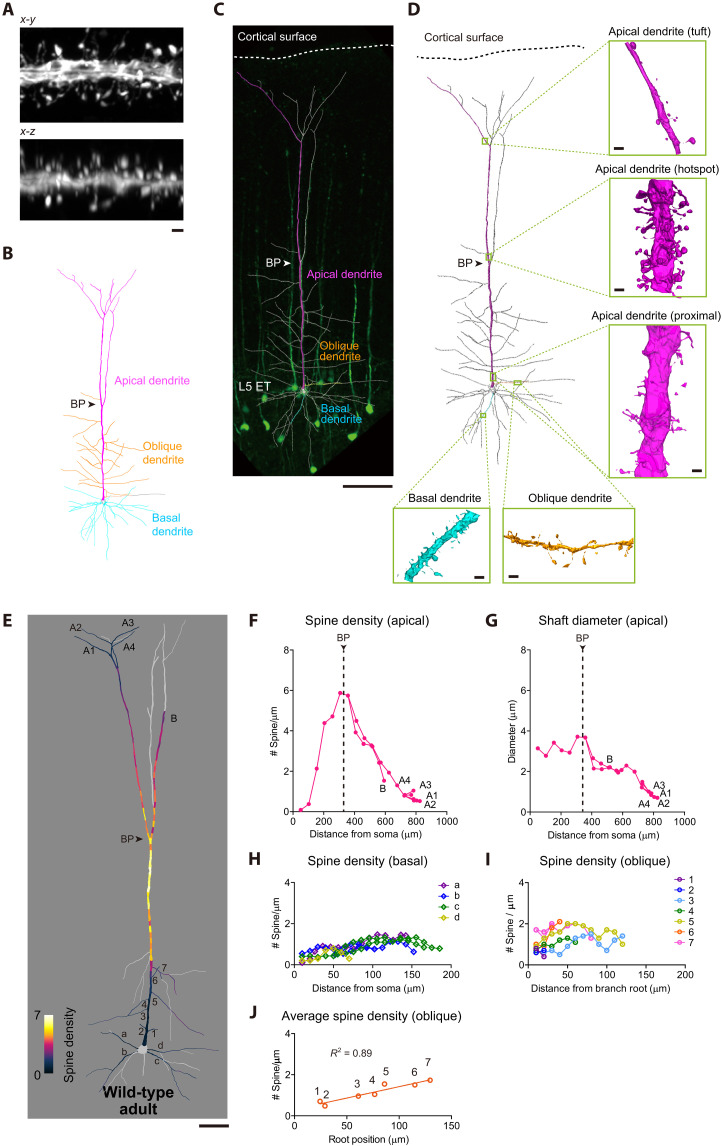
Whole-neuron scale super-resolution imaging and reconstruction of L5 ET neurons in S1. (**A**) Airyscan super-resolution imaging of dendritic spines (apical dendrites of L5 ET neurons, adult Thy1-YFP-H line, age P56). Brain slices were cleared with SeeDB2S. Resolution in ~150-nm lateral resolution and ~300-nm axial resolution was sufficient to unambiguously identify all the dendritic spines in three-dimensional. Scale bar, 1 μm. (**B**) Schematic representation of L5 ET neurons. BP, bifurcation point. (**C**) A L5 ET neuron of a Thy1-YFP-H mouse (age P56) was imaged with Airyscan with 63× objective lens (NA 1.4, WD 0.19 mm). See also fig. S1 for schematic workflow. Scale bar, 50 μm. (**D**) Representative segments from apical, basal, and oblique dendrites were reconstructed with VAST Lite. Scale bars, 1 μm. BP, bifurcation point. (**E**) Spine density map (spine number/μm, plotted every 10 μm) for another representative L5 ETneuron (Thy1-YFP-H, age P63). Dendritic segments found within the working distance of the 63× objective lens were analyzed. Pale gray dendrites were outside the working distance. See also fig. S2 and movie S1 for super-resolution images of apical dendrite. (**F**) Spine density per dendritic length (spine number/μm, plotted every 50 μm) in apical dendrites. All of the branches within the working distance were traced (terminal A1 to A4 and F). BP, bifurcation point. (**G**) Dendritic shaft diameter for the corresponding segments in (F). (**H**) Spine density in basal dendrites. Four basal dendrites [a to d shown in (E)] are shown in different colors. (**I**) Spine density in oblique dendrites. Seven oblique dendrites [#1 to 7 shown in (E)] were analyzed. (**J**) Root position–dependent variations of spine density in oblique dendrites. Root positions in apical dendrites (distance from the soma) and average spine densities were determined for oblique dendrites #1 to 7. *R*^2^ (coefficient of determination) = 0.89, *P* < 0.01.

To accurately map synapses, electron microscopy (EM)–based connectomics is the gold standard (*27*). However, the annotation of massive EM images at a millimeter scale is still laborious and difficult. Conventional light microscopy did not have sufficient resolution to identify all synapses; its resolution is particularly poor along *z* axis. To overcome this issue, we have previously developed a tissue clearing agent, SeeDB2, designed to minimize spherical aberrations and enable super-resolution fluorescence imaging of thick brain samples (*28*). Our strategy allows for large-scale imaging with a constant spatial resolution of ~150 nm in *x*-*y* and ~300 nm in *z*, which is sufficient to quantify the number and size of dendritic spines accurately (Fig. 1A and fig. S1).

In the present study, we performed comprehensive super-resolution spine mapping for layer 5 extratelencephalic-projecting (L5 ET) neurons (also known as thick-tufted L5 neurons and subcerebral projection neurons in layer 5b) in the primary somatosensory cortex (S1) during postnatal development in mice. We found that highly spine-rich areas (spine density “hotspot” with ~10-fold higher density) are formed at the middle compartment of the apical dendrites in an activity-dependent manner during adolescence. We also found that the spine accumulation at the hotspot during adolescence is specifically impaired in schizophrenia models in mice, suggesting that the compartmentalized regulation of spine density is critical for establishing higher cognitive functions during adolescence.

## RESULTS

### Large-scale super-resolution spine mapping using SeeDB2

In the present study, we performed a comprehensive super-resolution mapping of dendritic spines using SeeDB2, a tissue clearing agent optimized for high numerical aperture (NA) objective lenses with oil or glycerol immersion. Because of minimized spherical aberrations, we could obtain ~150-nm resolution in *x*-*y* and ~300-nm resolution in *z* for >100-μm-thick brain slice samples using commercialized super-resolution imaging systems (Airyscan from Carl Zeiss and HyVolution/LIGHTNING from Leica) (Fig. 1A and fig. S1) (*28*). Our strategy is more efficient than volume EM and more accurate than conventional light microscopy (e.g., confocal microscopy and two-photon microscopy), allowing accurate spine mapping at unprecedented scale and throughput.

We focused on L5 ET neurons in the whisker region of the somatosensory cortex (S1) as a model system, as they are one of the best characterized neuronal subtypes with distinct types of dendrites (*24*, *25*, *29*–*31*). We used the Thy1-YFP-H transgenic line which preferentially labels L5 ET neurons in the cortex (*32*). Among the labeled neurons, L5 ET neurons can be easily identified based on their large soma size and dendritic morphology. A typical L5 ET neuron has a long apical dendrite that extends toward L1 and basal dendrites that extend within L5 (Fig. 1B). The terminals of apical dendrites are ramified in L1, referred to as dendritic tufts. In addition, the proximal trunk region of an apical dendrite extends several oblique dendrites in horizontal directions. L5 ET neurons are known to integrate bottom-up inputs from the sensory thalamus at basal dendrites and top-down inputs from higher cortical regions at apical tufts and to mediate cortical output for sensory perception, thus serving as gatekeepers for corticofugal output (*11*, *33*, *34*).

To perform comprehensive spine mapping, we first obtained low-resolution images using a 20× (NA = 0.80, working distance (WD) = 0.55 mm) objective lens (Fig. 1C). Then, we focused on one particular neuron and imaged its dendrites using super-resolution imaging with 63× objective lens (fig. S1 and S2 and movie S1). All the high-resolution images were then stitched for dendritic tracing and spine mapping with Neurolucida software (fig. S2). We examined spine distribution in the apical, basal, and oblique dendrites. In basal and oblique dendrites, the spine density was relatively uniform as has been described in many previous studies; however, there was a highly skewed spine distribution in apical dendrites with thick dendritic trunk, which has been difficult to resolve with conventional light microscopy (Fig. 1D) (*35*).

### Spine density is highly skewed in apical dendrites of L5 ET neurons

**Fig. 2.**
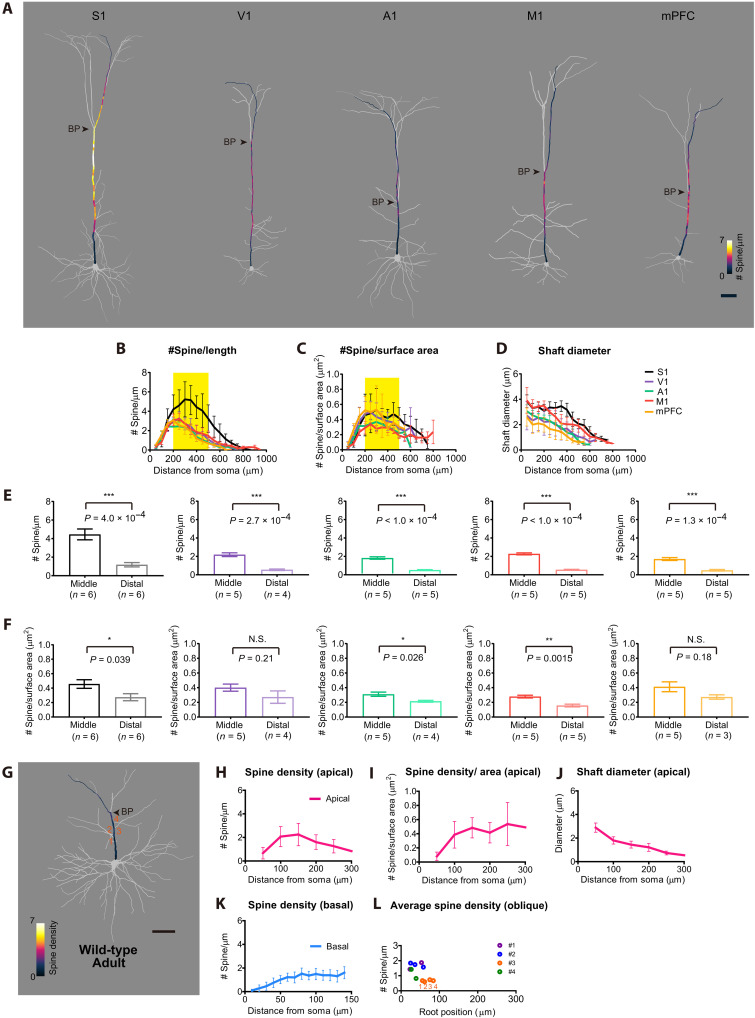
Spine density distribution in other cortical areas and cell types. (**A**) Spine density maps (every 10 μm) for representative L5 ET neurons in primary somatosensory (S1), primary visual (V1), primary auditory (A1), primary motor (M1), and medial prefrontal (mPFC) cortices in the Thy1-YFP-H mice. Scale bar, 50 μm. (**B**) Spine density per dendritic length every 50 μm. The peak position was slightly different across areas, possibly reflecting the cortical thickness (*37*). *n* = 5 dendrites each from three mice (age P77 to P84). The middle compartment (200 to 500 μm from soma) is highlighted. See also fig. S4A. (**C**) Spine density per dendritic surface area (spine number/μm^2^) along apical dendrites every 50 μm. The middle compartment (200 to 500 μm from soma) is highlighted. (**D**) Dendritic shaft diameter along apical dendrites every 50 μm. (**E**) Spine density per dendritic length in the middle (200 to 500 μm) and distal (>500 μm) compartments of apical dendrites. See also fig. S4A. (**F**) Spine density per surface area in the middle (200 to 500 μm) and distal (>500 μm) compartments of apical dendrites. See also fig. S4B. (**G**) A L2/3 neuron in S1 labeled by in utero electroporation (age, P77). The numbers indicate oblique dendrites. Scale bar, 50 μm. (**H**) Spine density per dendritic length (spine number/μm) in apical dendrites (every 50 μm). *n* = 5. (**I**) Dendritic shaft diameter (every 50 μm). See also fig. S4 (C and D). (**J**) Spine density per dendritic surface area (spine number/μm^2^). (**K**) Spine density in basal dendrites. *n* = 9. (**L**) Spine density in oblique dendrites. Each color indicates dendrites from the same neuron (*n* = 4 neurons). *R*^2^ = 0.9993 (#2, *P* = 0.0165), and 0.1907 (#3, *P* = 0.5633). See also fig. S3. Data are means ± SD. *P < 0.05, **P < 0.01, and ***P < 0.001 (Student’s *t* test). N.S., not significant.

We therefore quantified spine density in apical dendrites, from their proximal to distal parts in full (fig. S2). The spine density per dendritic length (micrometer) was determined and mapped onto the reconstructed dendrites (Fig. 1E). In the proximal part of apical dendrites, there was an aspiny region, and then the spine density gradually increased. The highest spine density was found at the middle compartment of apical dendrites, near the first bifurcation point (Fig. 1F and movie S1). In the more distal part of apical dendrites, the spine density was lower. The spine density around the first bifurcation point (5.6 ± 1.7 spines/μm) was over 10-fold higher than the lowest area (<0.5 spines/μm) (see also Fig. 2B). Notably, the spine density found in this study was >5 times higher than previous studies using the conventional light microscopy (*35*), suggesting that improved resolution was critical for accurate quantification of spines. The diameter of the apical dendrite alone cannot explain the skewed spine density at this location (Fig. 1G). In contrast, spine density was less biased in the basal dendrites, although the proximal regions tended to have fewer spines (Fig. 1H). In the oblique dendrites, the spine density was almost uniform along the entire length of the dendrites (Fig. 1I). However, when we compared different oblique dendrites originating from different proximal-distal locations of the same apical dendrite, the spine density was different. Oblique dendrites originating from more proximal locations along the apical dendrite showed a lower spine density, while those originating from more distal positions tended to have more spines (Fig. 1J). In other words, the spine density of oblique dendrites reflects that of the root position in apical dendrites. Thus, the apical trunk region near the first bifurcation point is a specialized dendritic compartment.

We examined whether the spine density is correlated with a specific layer or the geometry of the L5 ET neurons. There is a considerable variation in the first bifurcation point of apical dendrites among L5 ET neurons (*30*). The bifurcation point is important because it integrates inputs from different dendrites, which can lead to large dendritic spikes known as Ca^2+^ spikes (Ca^2+^ plateau potentials) with voltage-gated Ca^2+^ channels (*36*). We used anti-VGluT2 antibody to label thalamic axon terminals in the barrel structure in layer 4 (fig. S3, A and B). We found that the location of the highest spine density was correlated with the location of the first bifurcation point (fig. S3, C to E).

### The skewed spine density in other areas and cell types

To examine whether the highly skewed spine density in apical dendrites is a general feature of L5 ET neurons, we also analyzed the spine distribution for L5 ET neurons in primary visual cortex (V1), primary auditory cortex (A1), primary motor cortex (M1), and medial prefrontal cortex (mPFC). Cortical thickness was variable in the different areas (*37*). Correspondingly, the length, shaft diameter, and spine density in apical dendrites varied across areas, with the highest in S1 (Fig. 2, A and B). Nevertheless, the spine density was highest at the middle compartment of apical dendrites in all areas (Fig. 2, A and B to E, and fig. S4). If synapses are formed according to the presynaptic density, then the spine density would be proportional to the surface area of the dendrites. However, we found that spine density per surface area in the middle compartment (~200 to 500 μm from the soma) is higher than in the distal compartment in the apical dendrites (Fig. 2, C and F; spine density hotspot).

We further examined whether the biased spine density of apical dendrites is a unique feature of L5 ET neurons. We examined the spine density of layer 2/3 (L2/3) neurons, another type of cortical pyramidal neuron. We labeled L2/3 neurons with tdTomato using in utero electroporation at embryonic day 15 (E15), and their morphology was analyzed at adult stage. Spine density mapping revealed only modest density biases both in their apical and basal dendrites (Fig. 2, G to L). Unlike L5 ET neurons, the first bifurcation point did not show higher spine density, and there was no difference in spine density between the middle and distal domains (fig. S4, C and D). Furthermore, oblique dendrites did not demonstrate a clear root position-dependent change in spine density (Fig. 2L). These results are consistent with a recent study focusing on L2/3 neurons (*38*). Thus, accumulation of the spines in the middle compartment is prominent in the apical dendrites of L5 ET neurons but not in L2/3 neurons.

### Spine density at the hotspot specifically increases during adolescence

**Fig. 3.**
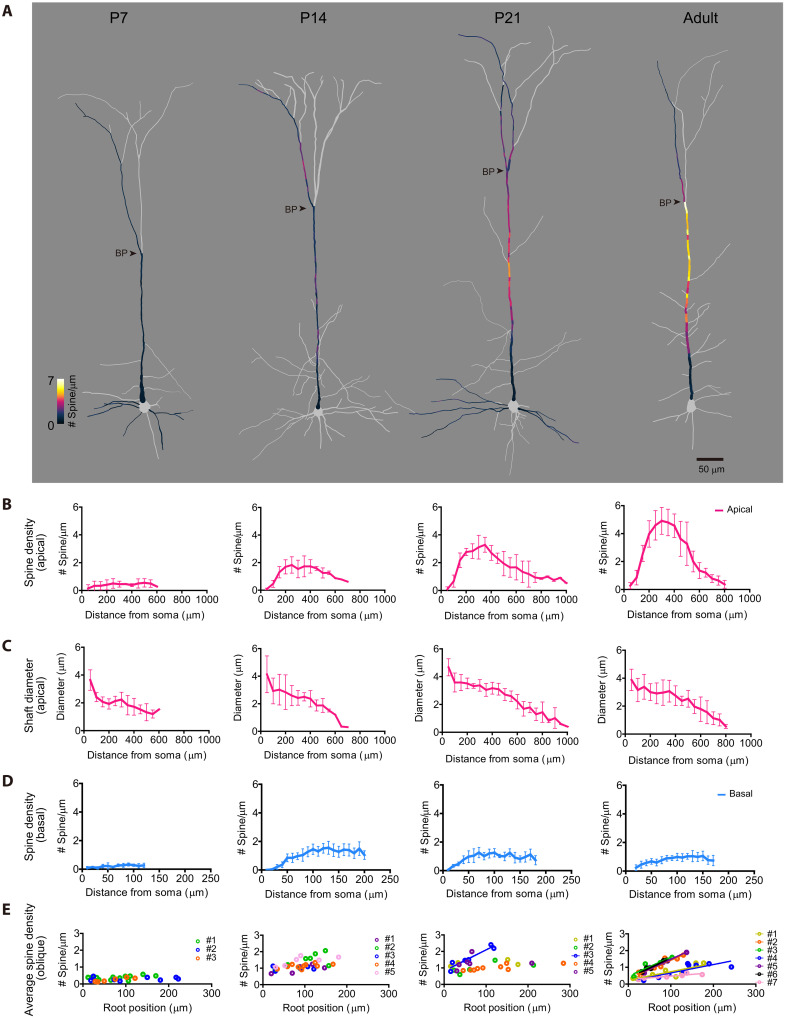
Dynamic spine density changes during adolescent cortical development. (**A**) Spine density mapping for L5 ET neurons at P7, 14, 21, and adult (P76) (S1 in Thy1-YFP-H mouse line). Representative neurons are shown. Spine density (determined every 10 μm) is indicated by color code. Scale bar, 50 μm. (**B**) Spine density per dendritic length (spine number/μm) was plotted along apical dendrites. Data are from four (P7), five (P14), five (P21), and five (adult, age P63 to P84) neurons. Data are means ± SD. (**C**) Dendritic shaft diameter for the corresponding dendritic segments shown in (B). Data are means ± SD. (**D**) Spine density in basal dendrites. Data are from 4 (P7), 8 (P14), 11 (P21), and 9 (P56 to P84) dendrites. Data are means ± SD. See also fig. S5 (A to E) for individual basal dendrite from one representative neuron. (**E**) Spine density in oblique dendrites. Root positions and average spine density in oblique dendrites were determined for multiple neurons. Each color indicates sibling oblique dendrites from the same neuron. Data are from three (P7), five (P14), six (P21), and seven (P63 to P84) neurons. Correlation coefficients were 0.19, 0.02, and 0.87 at P7; 0.36, 0.40, 0.01, 0.31, and 0.53 at P14; 0.31, 0.13, 0.90, 0.32, and 0.02 at P21; and 0.70, 0.89, 0.84, 0.72, 0.98, 0.97, and 0.76 in the adult. *P* = 0.16, 0.74, and 0.0071 at P7; 0.21, 0.092, 0.90, 0.094, and 0.10 at P14; 0.33, 0.37, 0.0037, 0.091, and 0.80 at P21; 0.0093, 0.0014, 0.0010, 0.083, 0.0010, 0.00030, and 0.010 in the adult. Root position–dependent changes in spine density were only evident in the adult. See also fig. S5 (F to J) for individual oblique dendrite from one representative neuron.

Next, we examined how the biased spine density is formed during development. We performed spine mapping in apical, basal, and oblique dendrites at postnatal day 7 (P7), P14, P21, and adult (P56 to P84) (Fig. 3A and fig. S5). At P7, spine density was low and the distribution was almost uniform. Between P7 and P14, the spine density, as well as the dendritic diameter, increased in all compartments of the dendrites except for the very proximal part of dendrites. Between P14 and the adult, spine density in basal dendrites slightly reduced as has been well known (*5*, *8*, *35*) (Fig. 3D); in contrast, spine density in the middle part of the apical dendrites continued to increase during adolescence (Fig. 3B and fig. S3F). Spine density per dendritic surface area in the middle compartment was significantly higher than in the distal part in the adult, but not at P14 (fig. S3, G and H). Oblique dendrites also demonstrated their root position-dependent spine density changes during adolescence (Fig. 3E). Thus, the prominent spine density hotspot emerges during adolescence.

**Fig. 4.**
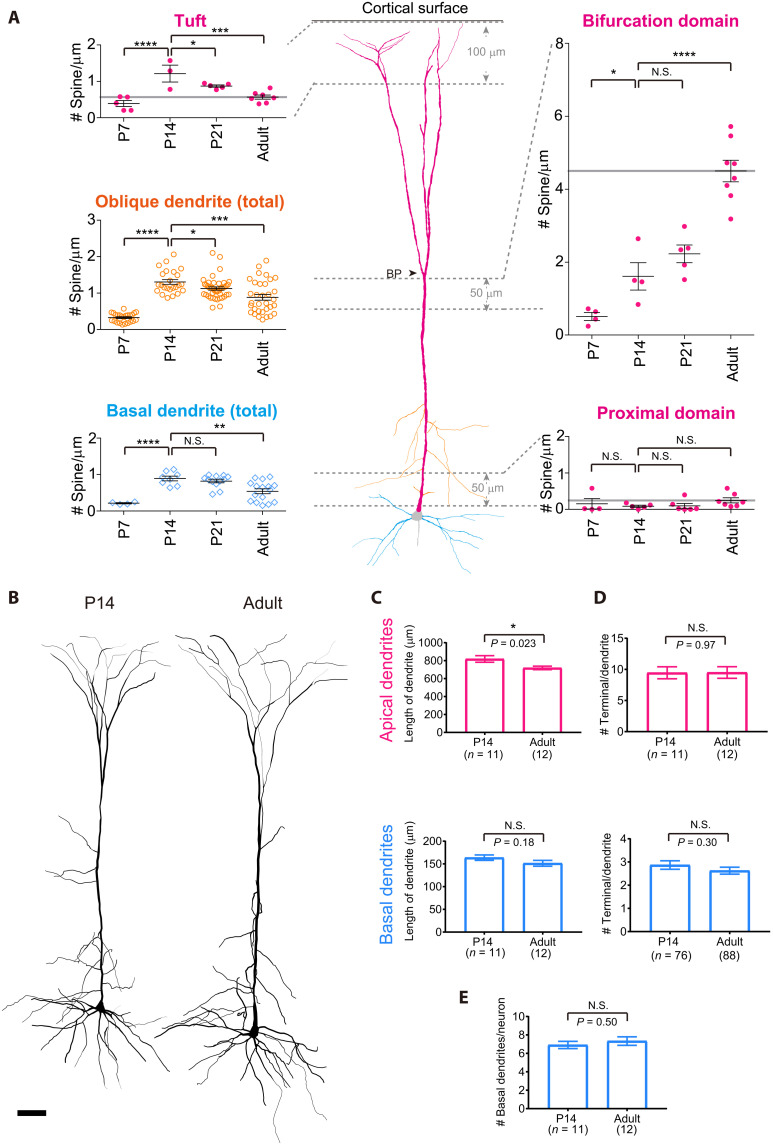
Dendritic compartment-specific regulation of spine density during development. (**A**) Average spine density in different dendritic compartments. Spine density was averaged for full length in basal and oblique dendrites. The proximal domain of apical dendrites was defined as a 50-μm region just adjacent to the most proximal a spiny region. The bifurcation domain was defined as a 50-μm region of the apical trunk just below the first bifurcation point (see also fig. S3E). The tuft domain was defined as an area within 100-μm depth from the pia surface. Black lines indicate means ± SEM, and gray horizontal lines indicate average spine density in the adult. Data are means ± SD. Data are from 5 (P7), 3 (P14), 5 (P21), 7 (P63 to P84) tufts; 4 (P7), 4 (P14), 5 (P21), and 8 (P63 to P84) bifurcation domains; 4 (P7), 5 (P14), 6 (P21), and 7 (P63 to P84) proximal domains; 4 (P7), 8 (P14), 13 (P21), and 16 (P56 to P84) basal dendrites; 23 (P7), 21 (P14), 40 (P21), and 40 (P63 to P84) oblique dendrites. Spine density was compared between P14 and other ages. *P < 0.05, **P < 0.01, ***P < 0.001, and ****P < 0.0001 (Benjamini-Hochberg method compared with the P14). (**B**) Dendritic patterns of representative L5 ET neurons at P14 and adult (P84). Scale bar, 50 μm. (**C**) The average length of apical (top) and basal dendrites (bottom). (**D**) Number of dendritic terminals per dendrite for apical (top) and basal (bottom) dendrites. (**E**) Number of basal dendrites originated from the soma of an L5 ET neuron. Data are means ± SEM. Data are from 11 (P14) and 12 (adult) apical dendrites; 76 (P14) and 88 (P77-84) basal dendrites from 11 (P14) and 12 (adult) neurons in each 3 animals. *P < 0.05 (Student’s *t* test).

Developmental changes in spine density were highly skewed even within apical dendrites. We determined the spine density of different compartments in apical dendrites: the most proximal part (the first 50-μm segment adjacent to the aspiny region), the bifurcation domain (50-μm segment just before the first bifurcation point), and the tuft (dendritic segments within 100-μm depth from the cortical surface) (Fig. 4). While the bifurcation domain continued to increase spine density during adolescence, the tuft region of the apical dendrites demonstrated a moderate reduction after P14, as has been reported (Fig. 4A) (*39*). We found no differences between males and females (fig. S3I). We did not find any changes in aged animals (fig. S3J). Thus, among the different dendritic compartments in L5 ET neurons, only the middle compartment of apical dendrites demonstrates spine accumulation during adolescence. All other compartments undergo a moderate reduction in spine density (Fig. 4A).

A reduction in spine density may be due to more spines being eliminated than formed (*39*). Alternatively, it may be due to dendritic elongation without changing the total number of spines. We found that the overall dendritic morphology of L5 ET neurons does not change during adolescence (Fig. 4B). We confirmed no increase in dendritic length (Fig. 4C) and number of branches (Fig. 4D) between P14 and the adult (P77 to P84). Therefore, the total number of synapses in basal dendrites should be reduced, most likely due to more spine elimination. The spine number increases only in the middle compartment (hotspot) of the apical dendrites.

### Sensory experiences are essential for adolescent spine accumulation at the hotspot

**Fig. 5.**
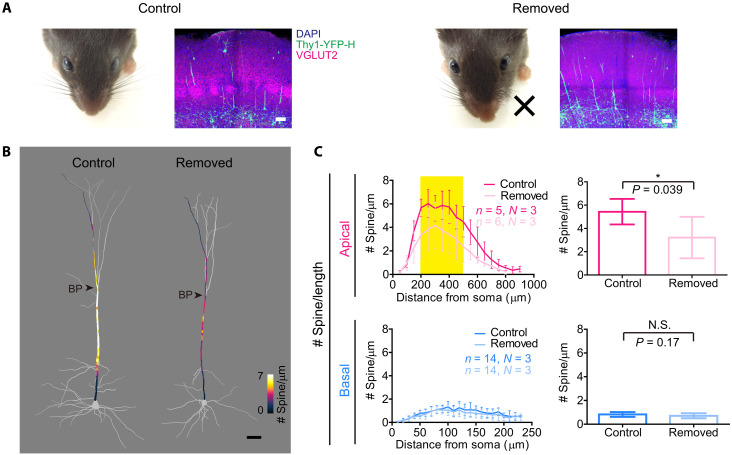
The spine density hotspot is formed in an activity-dependent manner during adolescence. (**A**) Whiskers on the left side were removed at P5. Regenerated whiskers were removed at P28 and P56. S1 sections on the right hemisphere were immunostained with anti-VGLUT2, a marker for thalamic axons. Control (left) and whisker-removed (right) mice are shown. Scale bars, 100 μm. (**B**) Spine density mapping for L5 ET neurons at P77 (S1 in Thy1-YFP-H mouse line). Spine density (determined every 10 μm) is indicated by the color code. Scale bar, 50 μm. (**C**) Spine density (per dendritic length, spine number / μm) in apical and basal dendrites after whisker removal. Data are from the contralateral hemisphere (right side) corresponding to the removed whiskers. Nontreated mice were used as controls. Mean spine density at the middle compartment of apical dendrites (200 to 500 μm from soma, highlighted in yellow) and basal dendrites are shown at the right. *n*, number of dendrites. Data are from five neurons each (adult, age P77). Data are from five (control) and six (removed) L5 ET neurons (each from 3 mice). Mice were P77. Data are means ± S.D. *P < 0.05 (Student’s *t* test).

As the spine density is specifically increased at the middle compartment of apical dendrites (hotspot) during adolescence, we investigated the underlying mechanisms. The hotspot near the first bifurcation point of apical dendrites is known to be highly electrogenic and capable of generating dendritic calcium spikes (*26*, *36*). Therefore, we considered the possibility that experience-dependent mechanisms play an important role in spine accumulation at the hotspot. The development of cortical circuits requires both spontaneous and evoked activity, but the role of spontaneous activity is limited during the early postnatal period (*40*–*42*). Therefore, we investigated the role of sensory experiences. We removed all the whiskers unilaterally from P5 (Fig. 5A). We found that spine density in basal dendrites was unchanged; however, spine density at the middle compartment of apical dendrites became significantly lower by the whisker removal (Fig. 5, B and C).

We, therefore, examined the role for *N*-methyl-d-aspartate (NMDA) receptors (NMDARs). We initially performed a single-cell knockout (KO) of *Grin1*, which encodes an essential subunit of NMDA receptors, GluN1, using in utero electroporation at E12 (*43*). We observed a reduction in spine density (fig. S6) and spine sizes (fig. S7, A and B). However, *Grin1*-deficient neurons also exhibited shorter and thinner dendrites, making their interpretation difficult. *Grin1* is known to be important for activity-dependent dendritic patterning at earlier stages of development (*40*–*42*, *44*).

**Fig. 6.**
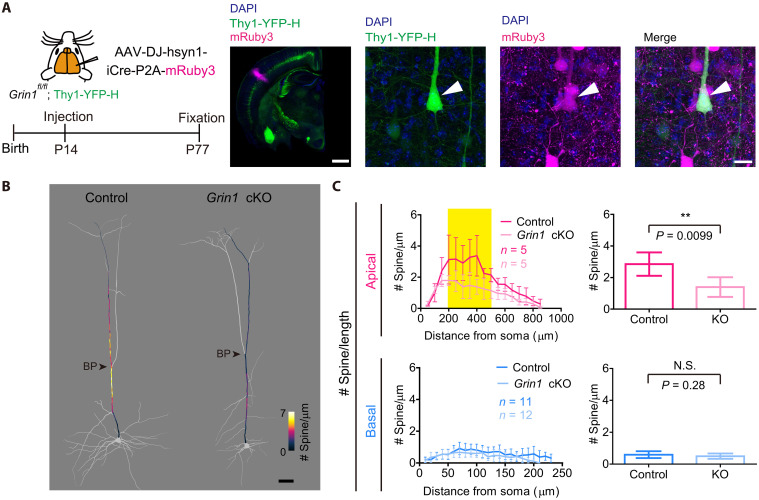
NMDARs are required for the hotspot formation during adolescence. (**A**) Age-specific *Grin1* cKO. AAV expressing Cre and mRuby3 was injected into *Grin1^+/+^* or *Grin1^fl/fl^; Thy1-YFP-H* mice at P14. Representative images of *Grin1*-deficient mRuby3^+^ and YFP^+^ L5 ET neurons (arrow). Scale bars, injection site overview, 1 mm; L5 ET neuron, 20 μm. (**B**) Spine density mapping for L5 ET neurons at P77 (S1 in Thy1-YFP-H mouse line) in control and adolescent *Grin1* cKO. Representative neurons are shown. Spine density (every 10 μm) is indicated by the color code. Scale bar, 50 μm. See also fig. S6 for *Grin1* cKO from embryonic stages. (**C**) Spine density per dendritic length (the number of spines/μm) in apical and basal dendrites in adolescent *Grin1*cKO. Mean spine density at the middle compartment of apical dendrites (200 to 500 μm from soma, highlighted in yellow) and basal dendrites is shown on the right. Spine density is slightly lower than in other experiments, even in the control, suggesting that AAV infection itself slightly suppressed spine formation. Data are from five (control) and five (cKO) L5 ET neurons (each from four mice). Mice were P77. Data are shown as means ± SD. **P < 0.01 (Student’s *t* test). See also figs. S7 to S9 for additional quantification data.

To specifically ablate *Grin1* during adolescence, we performed *Grin1* conditional KO (cKO) after P14. Adeno-associated virus (AAV) vectors carrying iCre and mRuby3 was injected to S1 region of *Grin1^fl/fl^* and *Grin1^+/+^* mice at P14, and L5 ET neurons (labeled in Thy1-YFP-H) positive for mRuby3 were analyzed at P77 (Fig. 6A). This time, we did not find differences in dendritic shaft diameter, suggesting that initial dendritic patterning was not affected (fig. S7A). In the basal dendrites, spine density was comparable between wild-type and *Grin1* cKO neurons. However, the spine density at the middle compartment of apical dendrites was significantly lower in *Grin1* cKO neurons (Fig. 6, B and C). Thus, NMDARs are essential for the adolescent spine accumulation to form the hotspot.

### Adolescent spine accumulation at the hotspot is impaired in schizophrenia models

As the spine accumulation at the hotspot was specifically impaired by sensory deprivation and adolescent *Grin1* cKO, we considered a possibility that its dysregulation is related to neuropsychiatric disorders. Notably, many neuropsychiatric disorders start to exhibit symptoms during adolescence (*45*). Schizophrenia is one of the most common neuropsychiatric disorders (~1% of the population) and is known to show symptoms during adolescence. In addition, patients with schizophrenia often experience sensory hallucinations, suggesting abnormalities in the sensory cortex, while the higher cognitive areas may also be affected. Human genomic studies suggested a link to several signaling pathways, including NMDARs, dopamine, and the immune functions (e.g., major histocompatibility complex) (*46*–*48*). Postmortem brains from patients with schizophrenia suggest reduced spine density (*8*, *10*). We therefore investigated whether the hotspot formation during adolescence is affected in mouse models of schizophrenia.

**Fig. 7.**
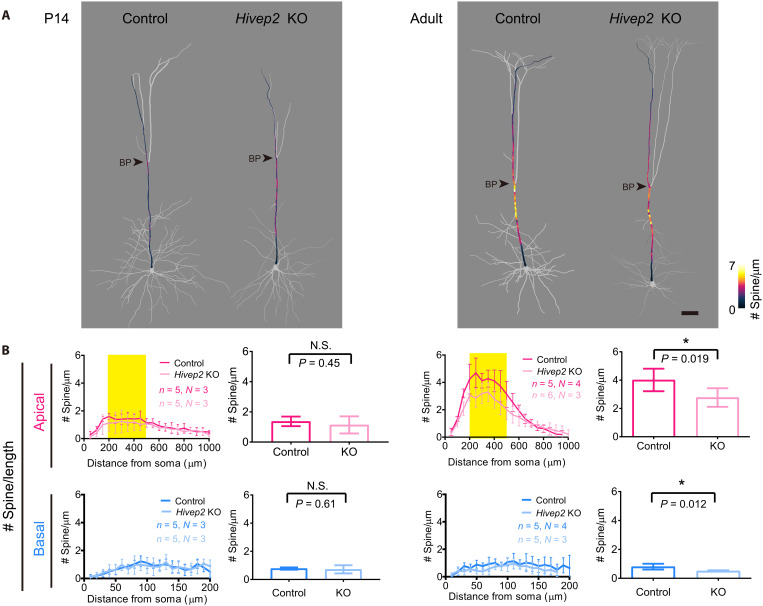
Hotspot formation is impaired in *Hivep2* KO mice. (**A**) Spine density mapping for S1 L5 ET neurons in *Hivep2* KO mice at P14 (left) and P77 (right) (Thy1-YFP-H mouse line). Spine density (every 10 μm) is indicated by the color code. Scale bar, 50 μm. (**B**) Spine density per dendritic length (spine number/μm) along dendrites is shown on the left. Right panels show the mean spine density at the middle compartment of apical dendrites (200 to 500 μm from soma, highlighted in yellow) and basal dendrites. Data are from five (control) and five (KO) L5 ET neurons (each from three mice) at P14. *N*, number of mice. Data are mean ± SD. *P < 0.05 (Student’s *t* test). See also figs. S7 to S9 for additional quantification data.

First, we studied *Hivep2* (also known as *Schnurri-2*, coding for a transcription factor) KO mice because *Hivep2* KO mice have been found to exhibit prominent behavioral abnormalities related to schizophrenia in the large-scale behavioral batteries (e.g., reduced prepulse inhibition and working memory) (*49*–*51*). Later studies in human showed that *HIVEP2* haploinsufficiency is linked to various neuropsychiatric impairment including intellectual disability (*52*–*54*). When we compared wild type and *Hivep2* KO, there was no clear difference in spine density at both basal and apical dendrites at P14. In the adult animals, however, the spine density was lower at both the middle compartment of apical dendrites and basal dendrites in the *Hivep2* KO (Fig. 7, A and B, and figs. S7, B and C and S8A). Thus, spine accumulation at the hotspot during adolescence was impaired in *Hivep2* KO.

**Fig. 8.**
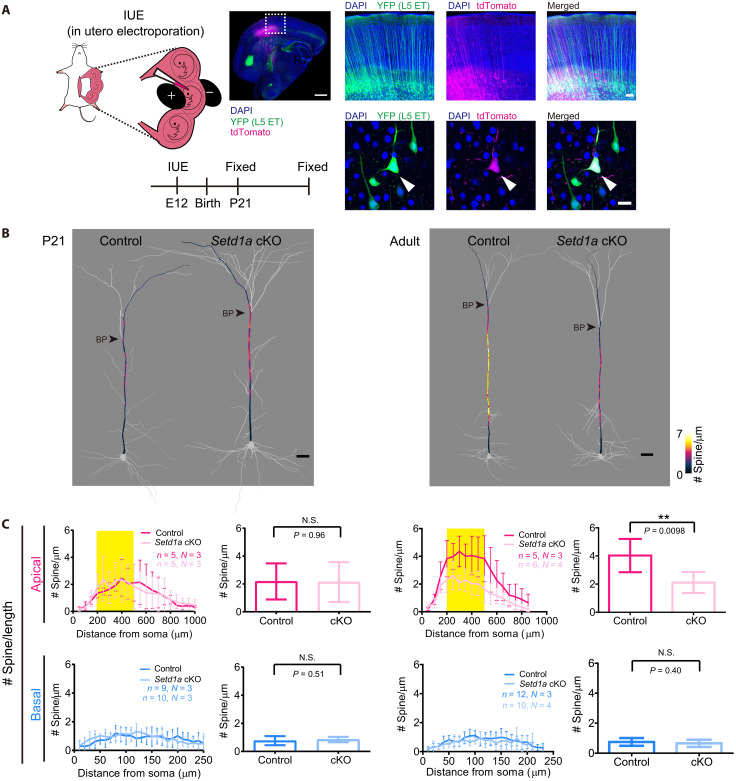
Hotspot formation is impaired in *Setd1a*-deficient neurons. (**A**) Schematic representation and schedule of in utero electroporation. CRISPR plasmids for *Setd1a* cKO (Cas9, 3× gRNAs, and tdTomato) were introduced into a subset of L5 neurons. Plasmids without *Setd1a* gRNA were used for the control. We examined tdTomato^+^ and YFP^+^ L5 ET neurons. A representative neuron is shown (arrowheads). Scale bars, 1 mm (top left), 100 μm (top right), and 20 μm. (**B**) Spine density mapping for S1 L5 ET neurons in *Setd1a* cKO neurons at P14 (left) and P77 (right) (Thy1-YFP-H mouse line). Spine density (determined every 10 μm) is indicated by the color code. Scale bar, 50 μm. (**C**) Left panels show spine density per dendritic length (spine number/μm) along dendrites. Right panels show the average spine density at the middle compartment of apical dendrites (200 to 500 mm from soma) and basal dendrites. Data are from five (control) and five (cKO) L5 ET neurons (each from three mice) at P21. Data are means ± SD. **P < 0.01 (Student’s *t* test). See also figs. S7 to S9 for additional quantification data.

Next, we examined another genetic mouse model of schizophrenia. Rare loss-of-function variants for *Setd1a* have been identified as a strong risk factor for schizophrenia (*47*, *55*, *56*). *Setd1a* gene encode histone 3 lysine 4 (H3K4) tri-methyltransferase and is required for neuronal function in a cell autonomous manner. *Setd1a* heterozygous KO mice recapitulated several behavioral abnormalities related to schizophrenia (*57*, *58*). We, therefore, generated sparse single-cell KO of *Setd1a* using in utero electroporation and CRISPR-Cas9 (*59*). When we analyzed P21 animals, we did not find clear differences in spine density at both basal and apical dendrites. In the adult, however, spine density in *Setd1a* cKO neurons was reduced at the middle compartment of apical dendrites (hotspot), but not in the basal dendrites (Fig. 8 and figs. S7, D and E and S8B). It should be noted that spine density increased between P21 and the adult in wild-type neurons (*P* = 0.046, Student’s *t* test), whereas it remained unchanged in *Setd1a* cKO neurons (*P* = 0.98, Student’s *t* test). Together, our results demonstrate that the reduced spine formation at the hotspot during adolescence, rather than the overpruning of spines, is a common phenotype between the two genetic schizophrenia models and adolescent *Grin1* cKO (see also fig. S9 for spine size).

### Functional correlates of the hotspot formation during adolescence

How is the hotspot formation related to the functional changes of L5 ET neurons? Upon glutamatergic synaptic inputs, dendrites generate local dendritic spikes with NMDARs, known as NMDA spikes (*60*). It has been hypothesized that the apical dendrites of L5 ET neurons produce large dendritic spikes, known as Ca^2+^ spikes (Ca^2+^ plateau potentials), based on the coincident synaptic inputs from apical tufts (L1) and basal dendrites (L5) (known as apical amplification hypothesis) (*24*, *25*). Ca^2+^ spikes are generated near the first bifurcation point of the apical trunk in an all-or-nothing manner with voltage-gated Ca^2+^ channels (*23*–*25*, *29*, *36*).

**Fig. 9.**
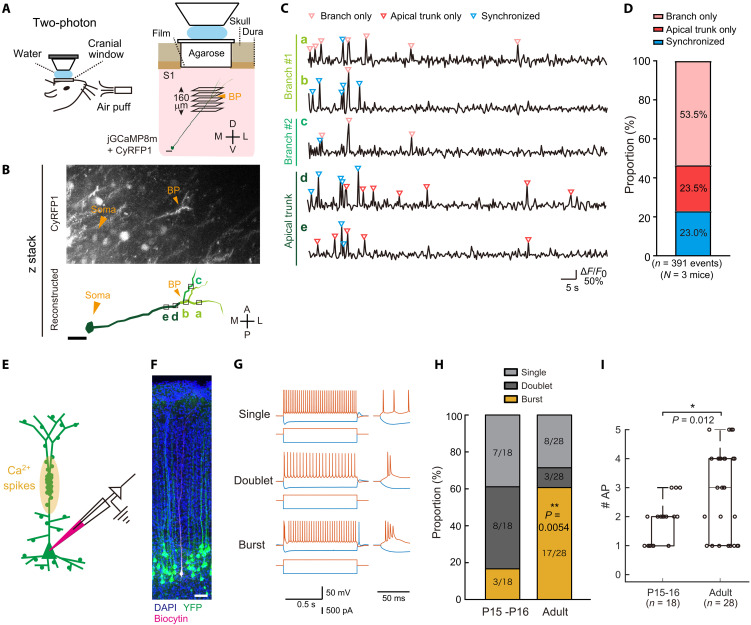
Functional roles of synaptic inputs to the hotspot. (**A**) Schematic representation of in vivo Ca^2+^ imaging in awake mice. L5 ET neurons were sparsely labeled with jGCaMP8m and CyRFP1 (filler). We imaged 160 to 240-μm-thick volume (five to seven planes/volume, 30-μm step) spanning the apical trunk (below the bifurcation point) and the sibling branches. D, dorsal; V, ventral; M, medial; L, lateral. Scale bar, 50 μm. (**B**) Reconstruction of an imaged neuron. BP, bifurcation point. The distance from soma was 507 μm (a), 420 μm (b), 454 μm (c), 419 μm (BP), 375 μm (d), and 306 μm (e). A, anterior; P, posterior; M, medial; L, lateral. Scale bar, 50 μm. (**C**) An example of jGCaMP8m signals (Δ*F*/*F*) at each part of dendrites of a 6-month-old mouse. The soma was located at a depth of 660 μm. We performed Ca^2+^ imaging at a depth of 250 to 410 μm. Regions of interest (ROIs) are indicated in (B). Each ROI was defined for dendritic shafts in each depth, reporting dendritic Ca^2+^ signals. Synchronized events (between apical trunk and sibling branches, cyan) and nonsynchronized events (pink and red) are indicated. See also fig. S10. (**D**) Proportions of synchronized and nonsynchronized events. (**E**) Schematic representation of the current injection experiment. (**F**) YFP^+^ L5 ET neurons were recorded. Scale bar, 100 μm. (**G**) Firing patterns of L5 ET neurons in P14 and adult (P64 to P82) mice upon current injection. Representative traces for single, doublet, and burst firing (≥3 spikes) are shown. (**H**) Proportion of cells that showed three types of firing after somatic current injection at P15 to P16 and adult (P64 to P82) (**P = 0.0054, Chi-squared test). (**I**) Number of action potentials (#AP) within the first 50 ms of the burst firing (nonbursting = 1). Data are median ± interquartile range. *P < 0.05 (Wilcoxon rank sum test). See also fig. S11.

We performed in vivo Ca^2+^ imaging of apical dendrites of S1 L5 ET neurons in awake animals. We imaged a volume spanning the apical trunk below the bifurcation point, where the hotspot is formed, and the sibling branches using a fast *z*-axis piezo drive (Fig. 9, A and B). We occasionally observed synchronized (neuron-wide) Ca^2+^ events, which presumably involve somatic activity (back-propagating action potentials and/or the large dendritic Ca^2+^ spikes such as plateau potentials) (fig. S10) (*17*, *61*). In addition, we often observed nonsynchronized (local) Ca^2+^ events, presumably dendritic NMDA spikes (generated by the activation of locally clustered spines independently of somatic action potentials): They were found not only in dendritic branches (branch only) but also in the trunk region of apical dendrites (apical trunk only), suggesting the hotspot receives independent synaptic inputs from their sibling dendritic branches (Fig. 9, C and D). We thus assume that independent sources of synaptic input to the hotspot contribute to the apical amplification and dendritic Ca^2+^ spikes based on coincident synaptic inputs to other dendritic compartments (i.e., basal dendrites and apical tufts).

We also examined whether dendritic Ca^2+^ spikes are developmentally regulated. Using current injection into the somata of L5 ET neurons, we examined generation of dendritic Ca^2+^ spikes in juvenile (P14 to P15) and adult animals: Bursting action potentials can be recorded when dendritic Ca^2+^ spikes are generated by voltage-gated Ca^2+^ channels in apical dendrites (*62*, *63*). We found that the dendritic Ca^2+^ spike is generated more efficiently in the adult than in juvenile animals (Fig. 9, E to H) (*64*). Membrane resistance and capacitance were unchanged between juvenile and adult mice (fig. S11). Thus, the formation of spine density hotspot is coupled to the emergence of dendritic Ca^2+^ spikes in this specific dendritic compartment.

## DISCUSSION

### Spine density regulation in L5 ET neurons during adolescence

In the present study, we found that spine density in L5 ET neurons is highly skewed: It was highest near the first bifurcation point in the apical dendrites. In our study, the spine density (per dendritic length) at the hotspot was ~5 spines/μm, an order of magnitude higher than that reported in previous studies using conventional confocal microscopy (*35*, *65*). However, the spine density found in our study was comparable to that found in a recent EM-based connectomic study of the same dendritic compartment (5.1 spines/μm) (*66*). Thus, the accuracy of our super-resolution spine mapping is comparable to that of the EM-based connectomics. Most likely, previous light microscopy studies have missed many of thin spines/filopodia and those extending along the axial direction at thick dendritic trunk due to poor spatial resolution, especially in *z* (*28*). Thus, the spine density may have been underestimated in many previous studies using light microscopy, especially in thick apical dendrites of L5 ET neurons (*35*).

**Fig. 10.**
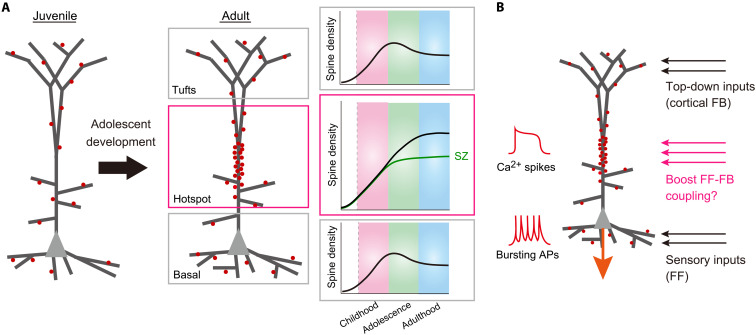
Proposed models for adolescent development and dendritic integration of L5 ET neurons. (**A**) A proposed model for the adolescent cortical development. Dendritic compartment specific regulation of spine density in L5 ET neurons is schematically illustrated. In basal dendrites, oblique dendrites, and the tuft region of apical dendrites, spine density increased during early childhood and then gradually decreased. In contrast, in the middle compartment of apical dendrites (hotspot), spine density continues to increase spine density during adolescence. Based on our developmental studies in mouse models, we propose that impaired spine formation during adolescence underlies cortical abnormalities in some (if not all) types of schizophrenia (SZ in green). Black lines indicate normal developmental trajectories. Spines are shown in red. (**B**) Possible role of synaptic inputs to the hotspot. The middle compartment of apical dendrites is known to generate dendritic Ca^2+^ spikes (Ca^2+^ plateau potentials) based on the coincident inputs to basal dendrites (sensory inputs, feedforward) and apical tuft dendrites (top-down inputs, cortical feedback). Thus, the synaptic inputs to the hotspot may boost the coupling of top-down (cortical feedback to layer 1) and sensory inputs (feedforward inputs to basal dendrites) to generate Ca^2+^ spikes, which are important for conscious sensory perception (*33*, *67*). APs, action potentials; FB, feedback; FF, feedforward.

In the present study, we also found that the spine density hotspot of L5 ET neurons increases during adolescence, while other dendritic compartments show a moderate reduction. Previously, developmental changes in synapse number have been considered in a stereotypical view: with an increase in synapse number during early childhood, followed by a decrease during adolescence to form mature cortical circuits (*8*, *9*). However, the spine density change is not stereotyped as has been previously considered. According to our study with L5 ET neurons in mouse S1, spine accumulation in the hotspot is another prominent change during adolescence (Fig. 10A).

### Roles of the spine density hotspot in apical dendrites

Our comprehensive spine mapping revealed that the spine density hotspot is formed only in the apical dendrites of L5 ET neurons, but not in their basal dendrites or in L2/3 neurons. Developmental analyses also showed that the spine density increases only in the apical dendrites during adolescence. What is special about this dendritic compartment in the adult?

It is known that the apical dendrites of L5 ET neurons produce large dendritic spikes with voltage-gated Ca^2+^ channels, known as Ca^2+^ spikes (Ca^2+^ plateau potentials), near the first bifurcation point (*24*, *36*). The dendritic Ca^2+^ spikes are critical for conscious perception and active sensing (*33*, *67*, *68*). The dendritic Ca^2+^ spikes in apical dendrites are also critical for the behavioral timescale synaptic plasticity (BTSP) (*69*). We confirmed that the dendritic Ca^2+^ spike is generated more efficiently in the adult ex vivo (Fig. 9, E to H). In one scenario, voltage-gated Ca^2+^ channels may accumulate developmentally and then spines are formed in an activity-dependent manner based on the Ca^2+^ spikes. Alternatively, it is also possible that voltage-gated Ca^2+^ channels are recruited to the hotspot based on the dense synaptic inputs to this region.

It has been proposed that L5 ET neurons integrate feedforward inputs from the sensory thalamus at basal dendrites and top-down feedback inputs at apical tufts to generate Ca^2+^ spikes, critical for generating bursting somatic action potentials and sensory perception (Fig. 10B) (*34*, *67*). The coupling of feedforward and feedback inputs is lost under anesthetized conditions, suggesting its role for consciousness (*33*, *70*). Previously, it has been considered that the apical dendritic trunk is aspiny and merely detect coincident inputs from the apical tuft and basal dendrites to generate the Ca^2+^ spikes with voltage-gated Ca^2+^ channels. However, our study revealed that dendritic spines are in fact highly enriched in this dendritic compartment (Figs. 1 and 2) receiving functional synaptic inputs (Fig. 9, A to D), raising the possibility that synaptic inputs to the hotspot is critical for generating and/or modulating the dendritic Ca^2+^ spikes. For example, the direct synaptic inputs to the hotspot may boost the coupling of feedforward and feedback inputs to generate the dendritic Ca^2+^ spikes (Fig. 10B). A recent study suggested that synaptic inputs to the apical trunk may facilitates the coupling of thalamocortical (to the basal dendrites) and corticocortical (to the distal apical dendrites) inputs, facilitating conscious perception (*33*).

Now, the origin of the synaptic inputs to the hotspot remains unclear. We assume that synaptic inputs to the hotspot is related to the sensory perception, active sensation, and/or consciousness (*33*, *67*, *68*). Future experiments should investigate the functional role of the direct synaptic inputs to the hotspot.

### Cortical development and sensory perception in schizophrenia models

Our understanding of schizophrenia is largely derived from human studies. Therefore, the cellular and circuit mechanisms of schizophrenia are still poorly understood. Synaptic density is reduced in some, if not all, types of schizophrenia based on the analyses of postmortem brains. Because symptoms appear after adolescence, it has been assumed that excessive spine elimination may underlie cortical dysfunction in schizophrenia (*8*–*10*). However, it is difficult to trace developmental trajectories in human studies. In this study, we examined developmental changes in spine distribution in multiple mouse models of schizophrenia (*Grin1* cKO, *Hivep2* KO, and *Setd1a* cKO) and found that experience-dependent spine formation during adolescence, rather than pruning, was commonly affected. Thus, we propose a model in which impaired “spine formation,” rather than overpruning, in specific dendritic compartments underlies cortical abnormalities in some (if not all) types of schizophrenia (Fig. 7I).

It is known that the Ca^2+^ spikes in apical trunk region (hotspot region) of L5 ET neurons is critical for sensory perception based on feedforward and feedback inputs (*34*). Sensory hallucinations, a major positive symptom in schizophrenia, and cognitive impairment, a major negative symptom in schizophrenia, may be due to dysregulation of this coupling process. It is possible that impaired spine formation at the hotspot leads to defects in dendritic integration and cognitive functions in schizophrenia. Conversely, impaired dendritic integration in schizophrenia could cause poor spine formation at the hotspot. In any case, it will be important to investigate how the dendritic integration is affected in L5 ET neurons during cognitive tasks in schizophrenia models.

A recent study reported another interesting feature in schizophrenia models: The formation of extra-large spines in L2/3 neurons in the PFC and bypassing of the synaptic integration process in these neurons (*71*). Together, the dysregulation of dendritic integration may be a key mechanism for cortical dysfunction in schizophrenia. The current study was mostly based on anatomical studies in mice. Given the human-specific mechanisms for dendritic integration (*34*, *72*), it will be important to explore which aspect of our findings are relevant to understanding neuropsychiatric disorders in humans.

## MATERIALS AND METHODS

### Mice

Mice expressing enhanced yellow fluorescent protein under the *Thy1* promoter, Thy1-YFP-H line (JAX, #003782) (*32*), were used for imaging L5 ET neurons. Floxed *Grin1* mice (*43*) and C57BL/6N mice were used for in utero electroporation. Both males and females were used for our imaging experiments. Ages are indicated in figure legends and table S1. All animal experiments were approved by the Institutional Animal Care and Use Committee of the RIKEN Kobe Branch (#AH-02-23) and Kyushu University (#A29-241, A19-070, A21-117, and A23-073). The *Hivep2* KO mouse has been reported previously (*73*). *Hivep2* KO mice with Thy1-YFP-H were obtained by breeding *Hivep2* heterozygous mice with Thy1-YFP-H in the C57BL6N background and *Hivep2* heterozygous mice in the BALB/cA background. For in utero electroporation, ICR mice crossed with male Thy1-YFP-H mice in the C57BL6/N background or C57BL/6N mice with Thy1-YFP-H were used.

### Plasmids

pCALNL-tdTomato (Addgene, #125573), pCAG-iCre (Addgene, #125574), pCAFNF-tdTomato (Addgene, #125575), pCAG-FLPo (Addgene, #125576), and pCAX-hCas9 have been reported previously. AiP1010-pAAV-mscRE4-minBGpromoter-iCre-WPRE-hGHpA (Addgene, #163476) was obtained from Addgene. Newly generated plasmids—such as pAAV-hsyn1-iCre-P2A-mRuby3 (Addgene, #235248), pAAV-CAG-FLEX-jGCaMP8m-P2A-CyRFP1 (Addgene #235249), and Setd1a gRNA #1-3 (Addgene, #235250-235252)—have been deposited to Addgene. Plasmids for *Setd1a* guide RNAs (gRNAs) were generated on the backbone vector. Three gRNA sequences that target different exons were designed as described previously (*59*). The sequences of the gRNAs are (5′-3′): *Setd1a* #1: TGGTCCGCTCTACGACCCG, *Setd1a* #2: AAGTCGATCCATAAGTTCC, and *Setd1a* #3: CGGCTCCACCAGTTACGGC.

### Optical clearing of mouse brain slices using SeeDB2

Mice were anesthetized with Nembutal and then intracardially perfused with 4% paraformaldehyde (PFA). Excised brains were fixed with 4% PFA at 4°C overnight and sliced with microslicer (Dosaka, PRO7N) at 220-μm thickness. PFA-fixed mouse brain slice samples were cleared with SeeDB2S as described previously (*28*). Slices were cleared in 1.5-ml tube with gentle shaking (~4 rpm) using a seesaw shaker at room temperature (25°C). First, fixed slice samples were incubated in 2% saponin in phosphate-buffered saline (PBS) overnight. Samples were incubated serially in 1:2 mixture of Omnipaque 350 and H_2_O with 2% saponin (solution 1) for >2 hours, followed by incubation in 1:1 mixture of Omnipaque 350 and H_2_O with 2% saponin (solution 2) for >2 hours. Samples were then incubated in Omnipaque 350 with 2% saponin (solution 3) for >2 hours. Last, samples were incubated in SeeDB2S [70.4% (w/w) iohexol dissolved in tris-EDTA solution] with 2% saponin for >12 hours. Cleared tissues were transferred to SeeDB2S mounting solution without 2% saponin for imaging and storage (Figs. 1, 3, and 4A, and figs. S1 to S5 and S7, A to C). PFA-fixed mouse brain sections sliced at 320-μm thickness were cleared with SeeDB2G as described previously (Fig. 2, 4B, and 5 to 8, and figs. S6 and S7, D to F). See also SeeDB Resources (https://sites.google.com/site/seedbresources/) for the step-by-step protocol.

### Immunohistochemistry

To facilitate antibody penetration, the samples were incubated in 2% saponin in PBS with gentle shaking (~4 rpm) for 24 hours at 4°C before blocking. The samples were then transferred to 1 ml of blocking buffer (0.5% skim milk, 0.25% fish gelatin, 2% saponin, 0.5% Triton X-100, and 0.05% sodium azide in PBS; in % w/v) in 1.5-ml tube. Blocking was performed with gentle rotation for 24 hours at 4°C. Reaction with primary antibodies (in 500-μl blocking buffer using 1.5-ml tube) was performed for 48 hours a seesaw shaker at room temperature. Guinea pig anti-VGluT2 (Millipore, AB2251) was used at 1:500 dilution. After three washes (2 hours each) in washing buffer (2% saponin and 0.5% Triton X-100 in PBS), samples were incubated with Goat anti-Guinea Pig secondary antibodies (Thermo Fisher Scientific, #A21435) at 1:500 dilution and 4′,6-diamidino-2-phenylindole solution (DOJINBO, #D523) at 1:1000 dilution in blocking buffer for 24 hours on a seesaw shaker at room temperature. Samples were then washed three times (2 hours each) in a washing buffer. The antibody-stained brain samples were then cleared with SeeDB2 as described above.

### Whisker removal

Whisker cauterization was performed as described previously (*74*). The whiskers on the left side of Thy1-YFP-H mice on the C57BL/6N background were removed with tweezers and cauterized under anesthesia at P5, P28, and P56. Mice at P5 were anesthetized with intraperitoneal injection of ketamine (90.9 mg/kg) and xylazine (9.08 mg/kg). Mice at P28 were anesthetized with intraperitoneal injection of ketamine (121 mg/kg) and xylazine (12.1 mg/kg). Mice at P56 were anesthetized with ketamine (40 mg/kg) and xylazine (7 mg/kg). Mice were anesthetized with overdose intraperitoneal injection of pentobarbital (Tokyo Chemical Industry, #P0776) and then perfused intracardially with 4% PFA. Excised contralateral brains were fixed with 4% PFA at 4°C overnight and sliced at 320-μm thickness. PFA-fixed mouse brain slices were cleared with SeeDB2G as described previously (*28*).

### AAV preparation

The AAVpro 293 T cell line (Takara Bio, #Z2273N) was cultured in 10-cm dishes. For the transfection, 6 μg of pHelper vector (Takara Bio, #6673), 6 μg of pAAV-DJ vector (Cell Biolabs, VPK-420-DJ), and 8 μg of pAAV vector of interest were mixed with 1.5 ml of Opti MEM solution (Thermo Fisher Scientific, #31985062) containing 60 μl of polyethylenimine MAX (Cosmo Bio). Plasmids were transfected to 60 to 80% confluent cells and cultured for 3 days. AAV was harvested using the AAVpro Purification Kit (Takara Bio, #6675). After purification, AAV was frozen in liquid nitrogen and stored at −80°C. To determine the titer, the AAV vector genome was extracted using the AAVpro Titration Kit (Takara Bio, #6233), and real-time PCR was performed on an Applied Biosystems 7500 Fast Real-Time PCR System (Thermo Fisher Scientific).

### AAV injection

Homozygous floxed *Grin1* mice (*43*) in C57BL/6N background were used to perform single-cell KO analyses of *Grin1*, which code GluN1, an essential subunit of NMDA receptor. Mice were anesthetized with an intraperitoneal injection of MMB [medetomidine (0.3 mg/kg), midazolam (4 mg/kg), butorphanol (5 mg/kg)] anesthetics (0.05 ml/10 g). The eyes were protected with Vaseline (FUJIFILM Wako, #227-01211), the head hair was cut, and the head was fixed with a stereotaxic (Narishige). The scalp was removed to expose the skull and a small hole was made with a dental drill (MINI PEN, Leutor). AAV-DJ-hsyn1-iCre-P2A-mRuby3 (6.9 × 10^12^ genome copy/ml) solution was injected into S1 (69 nL × 3 times) using Nanoject II and a glass capillary (#3-00-203-G/XL, Drummond). The holes in the skull were closed with Kwik-Sil (World Precision Instruments), and the scalp was sutured with surgical sutures and further glued with super glue (Daiichi Sankyo). After recovery from anesthesia with atipamezole hydrochloride injection (Kyoritsu Seiyaku) and on a hot plate, the mice were returned to their home cages.

### In utero electroporation

Homozygous floxed *Grin1* mice (*43*) in C57BL/6N background were used to perform single-cell KO analyses of *Grin1* coding for NMDAR1, an essential subunit for NMDA receptor function. pCALNL-tdTomato (1 μg/μl; Addgene, #125573) and pCAG-Cre (0.5 μg/μl; Addgene, #125574) were coelectroporated to the L5 pyramidal neurons at E12 and analyzed at P21 or P77. Wild-type C57BL/6N mice were used for labeling of L2/3 pyramidal neuron. The pCALNL-tdTomato (1 μg/μl) and pCAG-Cre (0.5 μg/μl) plasmids were coelectroporated to the L2/3 pyramidal neurons at E15 and analyzed at P77. In utero electroporation was performed as described previously using forceps-type electrodes (3- or 5-mm diameter) and a CUY21EX electroporator (BEX) (*28*). Pregnant ICR female mice crossed with homozygous Thy1-YFP-H mice in C57BL/6N background were used for single-cell KO analyses of *Setd1a*, which encodes SETD1A. *Setd1a* gRNAs (3×; 0.2 μg/μl each), pCAX-hCas9 (0.3 μg/μl), pCAFNF-tdTomato (1 μg/μl; Addgene, #125575), and pCAG-Flpo (0.05 μg/μl; Addgene #125576) were coelectroporated into L5 pyramidal neurons at E12, and mice were analyzed at P21 or P77.

### Super-resolution and confocal fluorescence microscopy

LSM880 with Airyscan (Carl Zeiss) was used for fluorescence imaging. Confocal images were acquired using a 20× dry objective (Plan-Apochromat 20×/0.8 M27, NA 0.80, WD 0.55) and GaAsP detectors. An 63× oil-immersion objective lens (Apochromat 63×/1.40 Oil DIC, NA 1.4, WD 0.19) was used for Airyscan super-resolution imaging. Fluorescence was detected under standard Airyscan mode, with an Airyscan detector and super-resolution mode [pinhole size, 1.25 Airy unit (AU)]. Enhanced yellow fluorescent protein (EYFP), tdTomato, and Alexa Fluor 647 were excited at 488, 561, and 633 nm, respectively.

In some experiments, Leica TCS SP8 (Leica) with a 10× dry objective (Leica HCX PL APO 10×/0.40CS, NA = 0.4, WD = 2.2 mm) and a 20× objective (Leica HC PL APO 20×/0.75 IMH CORR C52, NA = 0.75, WD = 0.68 mm) were used for confocal imaging. A 63× glycerol immersion objective lens (Leica HC PL APO 63×/1.3 Gly CORR C52, NA = 1.3, WD = 0.3 mm) was used for Lightning super-resolution imaging. Fluorescence was detected with HyD detectors. Lightning mode was used for the spine imaging (pinhole size, 0.6 AU). EYFP was excited at 488 nm. mRuby3 and tdTomato were excited at 552 nm.

For both Airyscan and Lightning, the *x*-*y* resolution was ~150 nm, and *z* resolution was 300 to 500 nm (full width at half maximum of fluorescence beads, 100 nm) (*28*).

### Slice electrophysiology

Thy1-YFP-H mice under C57BL/6N background were used for the slice electrophysiology. Recordings were performed at P15 to P16 or P64 to P82. Mice were deeply anesthetized with isoflurane and were intracardially perfused with an ice-cold cutting solution containing 210 mM sucrose, 2.5 mM KCl, 1.25 mM NaH_2_PO_4_, 8 mM MgCl_2_, 1 mM CaCl_2_, 26 mM NaHCO_3_, 20 mM glucose, and 1.3 mM sodium-l-ascorbate and bubbled with 95% O_2_ and 5% CO_2_ (*75*). The volume of solution perfused was 10 to 15 ml for P15 to P16 mice and 20 to 25 ml for P64 to P82 mice. Brains were immediately dissected, and coronal sections (300 μm) were cut using a vibratome (VT1200S, Leica). Slices were recovered in artificial cerebrospinal fluid (ACSF) at 32°C for 30 min and then maintained at room temperature for more than 30 min before recording. The slices were perfused with ACSF at 32° to 33°C during recording. The ACSF contained 126 mM NaCl, 3 mM KCl, 1.25 mM NaH_2_PO_4_, 1 mM MgCl_2_, 2 mM CaCl_2_, 26 mM NaHCO_3_, and 10 mM glucose and bubbled with 95% O_2_ and 5% CO_2_. Patch pipettes (3.4 to 5.3 MΩ) were filled with 130 mM potassium gluconate, 8 mM KCl, 1 mM MgCl_2_, 0.6 mM EGTA, 10 mM Hepes, 3 mM Na_2_ATP, 0.5 mM Na_2_GTP, 10 mM tris_2_-phosphocreatine, and 0.2% biocytin (pH 7.35). Whole-cell recording was performed for YFP^+^ L5 ET neuron somata in S1. Current injection was performed for 1 s. Recordings were performed using MultiClamp700B amplifiers (Molecular Devices), filtered at 10 kHz using a Bessel filter, and digitized at 20 kHz with Digidata 1440A digitizer (Molecular Devices). Data were stored using pClamp10 (Molecular Devices). The morphology of the recorded neurons was then visualized with streptavidin-Cy3 (#S6402, Sigma-Aldrich). Only L5 ET neurons with characteristic thick-tufted structure were analyzed.

### In vivo Ca^2+^ imaging of hotspots

We made a large cranial window with a polyvinylidene chloride (PVDC) wrapping film, silicone plug, and a coverslip as described previously (*76*, *77*). Briefly, mice were injected with 15% mannitol solution (3 ml/100 g body weight; Sigma-Aldrich, M4125) and anesthetized with isoflurane (induction: 2.5%, surgery: 0.7 to 1.3%). We made 3 mm–by–6 mm cranial window with dental drill over the right cortical hemisphere. Bleeding was stopped with a gelatin sponge. The dura was carefully removed. The brain surface was covered with a commercially available PVDC wrapping film (Asahi Kasei, Asahi Wrap or Saran Wrap, ~11-μm thick). The film was firmly attached to the skull with superglue. The coverslip (Matsunami 18 × 18 no. 1) was then sealed with a transparent silicone elastomer (GC Dental Products, Exaclear). jGCaMP8m was introduced to the primary somatosensory cortex (S1) using a combination of AAV vectors [AAV-PHP.eB-mscRE4-minBGpromoter-iCre-WPRE-hGHpA (*78*) and AAV-DJ-CAG-FLEX-jGCaMP8m-P2A-CyRFP1]. AAV-DJ-CAG-FLEX-jGCaMP8m-P2A-CyRFP1 (1.9 × 10^11^ gc/ml, 300 nl) was injected into C57BL/6N mice at P49 to P56 using the same method as AAV injection. AAV-PHP.eB-mscRE4-minBGpromoter-iCre-WPRE-hGHpA (2.6 × 10^10^ gc/ml, 100 ml) was injected through the right retro orbital.

Imaging was performed using a resonant scanning two-photon microscope (#MM201, Thorlabs) equipped with an objective piezo actuator [Physik Instrumente (PI), E-665 K007, or nanoFAKTUR, SF_-D20400] for fast *z*-axis control. A 920 nm femtosecond laser (ALCOR 920-4 Xsight, SPARK LASERS) and a 25× water-immersion objective lens (Olympus, XLPLN25XWMP2) were used for imaging. Whisker stimulation was applied via air puffs (2-s air puff every 15 s).

For Ca^2+^ imaging at the hotspots, the objective piezo actuator was used to acquire time-series data across a depth of 160 to 240 μm at 40 μm steps (five to seven planes/volume), which covered the hotspot and two sibling branches. The imaging was performed at 3 volumes/s (from the bottom to the top), with a time delay of 180 ms from the top to the bottom of the volume. This frame rate was sufficient to detect dendritic spikes accurately. Image data were analyzed with MATLAB (MathWorks). For peak detection, the “findpeaks” function in MATLAB was applied. Ca^2+^ “events” were defined as (i) peak Δ*F*/*F* > 50% and Δ*F*/*F* > baseline (five frames) + 3 SD or (ii) peak Δ*F*/*F* > 50%, and the peaks are detected in multiple regions of interest (ROIs). The small drifts were corrected by the Image Stabilizer plugin for ImageJ (https://imagej.net/plugins/image-stabilizer). ROIs were determined manually.

### Image processing and quantification

Neurolucida (MBF Bioscience) was used for manual neuronal tracing and quantification of dendritic spines in mouse L2/3 and L5 ET neurons. Analyses with Neurolucida were not blinded; instead, we performed comprehensive analyses. Quantitative data were exported from Neurolucida Explorer and processed with Microsoft Excel. Quantification of neuronal morphology was non-blinded. To avoid possible biases among samples, only cells with a typical pyramidal neuron morphology located within specific depth in somatosensory cortex were included in our dataset. For L2/3 neurons, brightly labeled neurons located 250 ± 50 μm beneath cortical surface were chosen for the analysis. For wild-type L5 neurons, EYFP-labeled neurons located 450 ± 50 μm beneath cortical surface were chosen for the analysis at P7 and P14; EYFP-positive neurons located 650 ± 50 μm beneath cortical surface were chosen for the analysis at P21 and adult. The spine density map is color coded using Morgenstemning (http://inversed.ru/Blog_2.htm).

VAST Lite (https://lichtman.rc.fas.harvard.edu/vast/) originally developed for serial EM images was used for reconstruction of super-resolution images in Fig. 1D. Traced image stacks were exported to MATLAB (MathWorks) to make surface meshes and then visualized with ParaView (www.paraview.org/).

### Statistical analysis

Statistical analyses were performed using GraphPad Prism 5. Correlations were calculated as Pearson’s correlation coefficient. Exact sample sizes are described in table S1. Numerical source data are summarized in table S2. Data are shown as means ± SD or means ± SEM. For the analyses of average spine density in different dendritic compartments, statistical differences were assessed by Benjamini-Hochberg method (Fig. 4A). For the analyses of dendritic length, statistical differences were assessed by Student’s *t* test based on near-normal distribution and similar variance (Fig. 4, B to E). For the analyses of mutant mice, statistical differences were assessed by Student’s *t* test based on near-normal distribution and similar variance (Figs. 5C, 6C, 7B, and 8C). Throughout this paper, *n* (small *n*) represents the number of dendrites (= number of neurons for apical dendrites) analyzed, and *N* (large *N*) represents the number of animals used in the analysis.
