# Supplementary material for: Dendritic compartment-specific spine formation in layer 5 neurons underlies cortical circuit maturation during adolescence
Source: Sci Adv. 2026 Jan 14;12(3):eadw8458. doi: 10.1126/sciadv.adw8458 (PMC12802838; doi:10.1126/sciadv.adw8458)
Supplement: Supplementary file 1 — Figs. S1 to S11 Legends for tables S1 and S2 Legend for movie S1 [file sciadv.adw8458_sm.pdf]

Science Advances
AAAS

Supplementary Materials for

Dendritic compartment-specific spine formation in layer 5 neurons underlies cortical circuit maturation during adolescence

Ryo Egashira *et al.*

Corresponding author: Takeshi Imai, imai.takeshi.457@m.kyushu-u.ac.jp

Sci. Adv. **12**, eadw8458 (2026)
DOI: 10.1126/sciadv.adw8458

The PDF file includes:

Figs. S1 to S11
Legends for tables S1 and S2
Legend for movie S1

Other Supplementary Material for this manuscript includes the following:

Tables S1 and S2
Movie S1

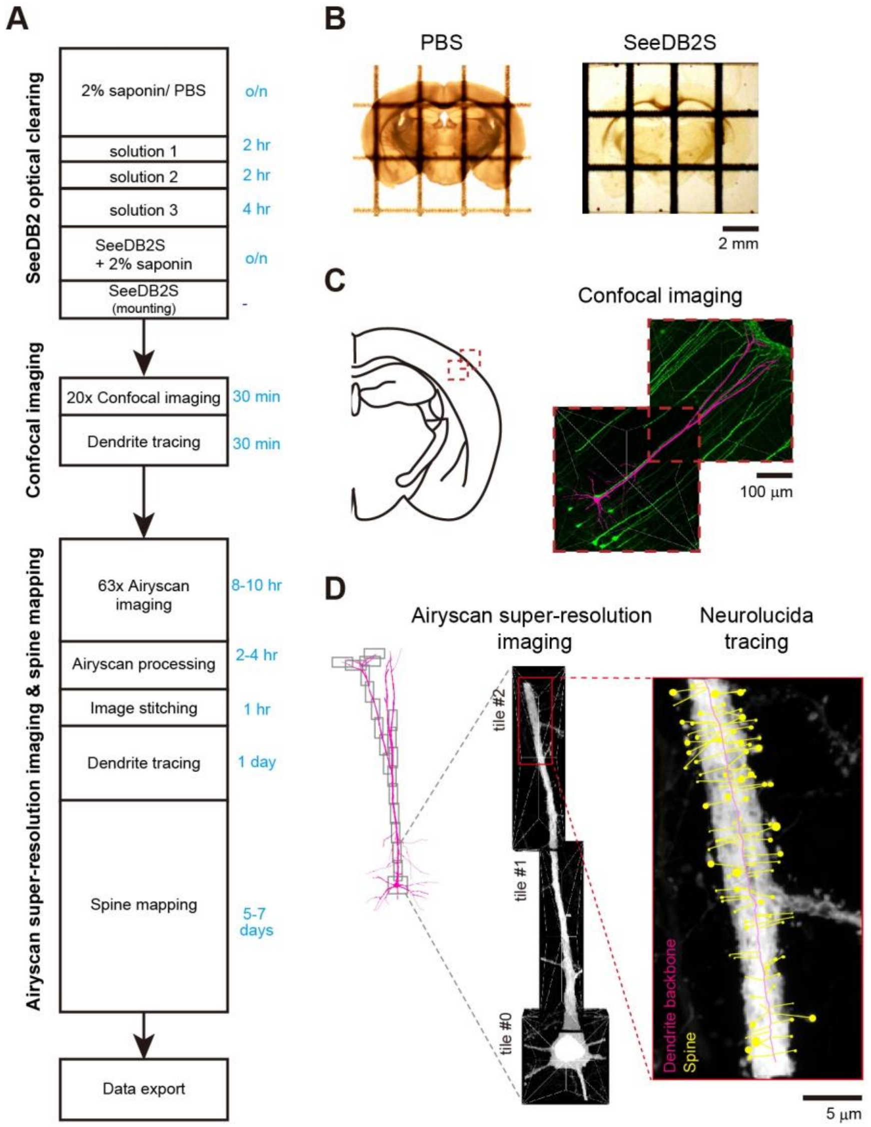

Fig. S1. Workflow for super-resolution spine mapping.

(**A**) Schematic workflow of our pipeline. We first prepared an optically cleared brain slice and then performed Airyscan (Zeiss) or LIGHTNING (Leica) super-resolution imaging for spine mapping. Brain slices are cleared with SeeDB2S according to the schedule shown in (**A**) and STAR METHODS.

(**B**) A 220 µm-thick adult brain slice before and after SeeDB2S clearing.

(**C**) The target neuron was first imaged with a 20x objective. Tiled images were then used for manual tracing with Neurolucida.

(**D**) Following the low resolution dendrite tracing, the full length of the target dendrite was imaged at the highest resolution using a 63x objective. The image shows a representative apical dendritic segment overlaid with the traced dendritic shaft and spines.

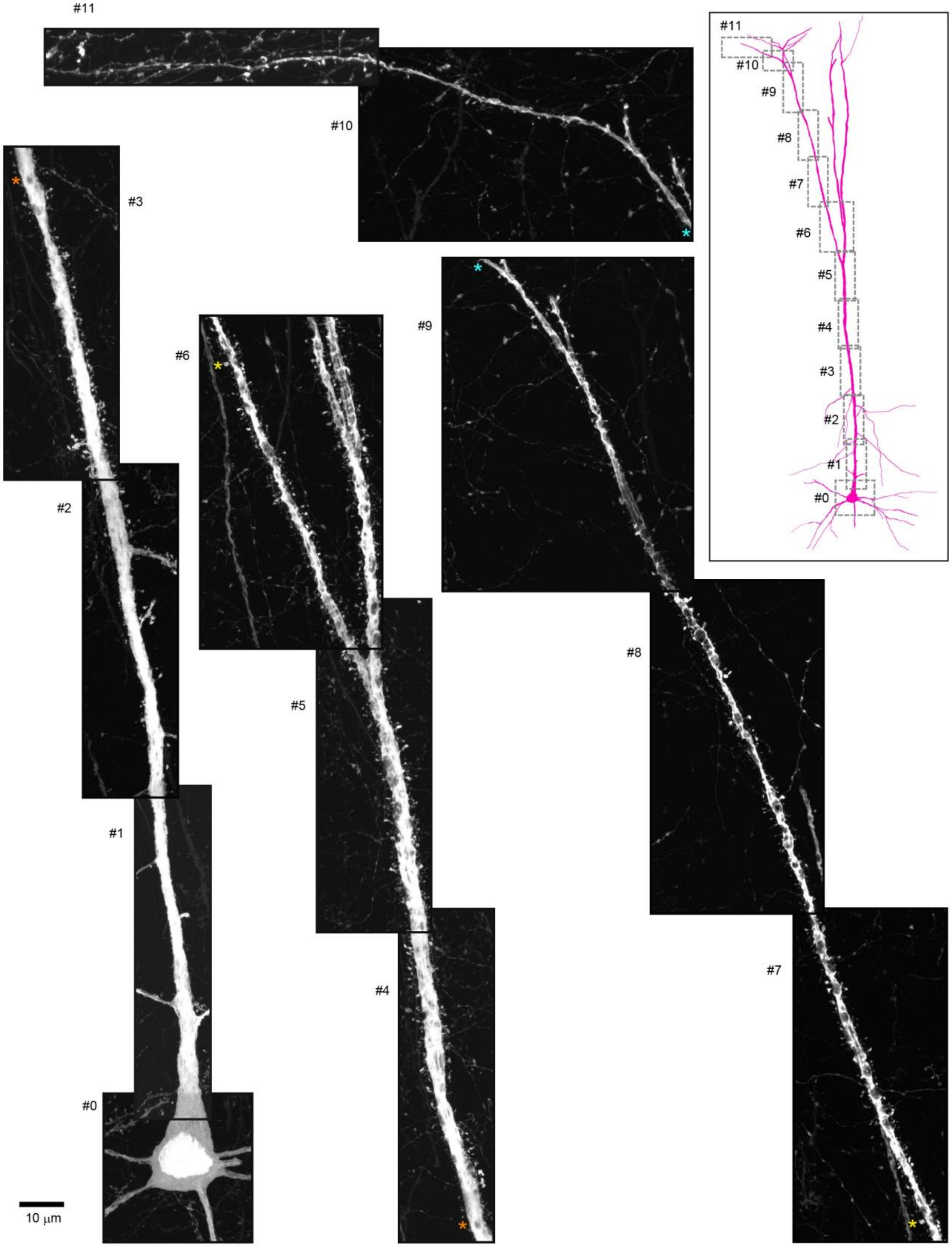

Fig. S2. Comprehensive super-resolution imaging of apical dendrite.

Airyscan super-resolution images of the full length of an apical dendrite of a thick-tufted L5 neuron (age, P63). An inset indicates the positions of 11 tiles. Asterisks (orange, yellow, and cyan) indicate the same locations in different tiles. Scale bar, 10 μm. See also **Movie S1** for a volume rendering of the entire length of the apical dendrite.

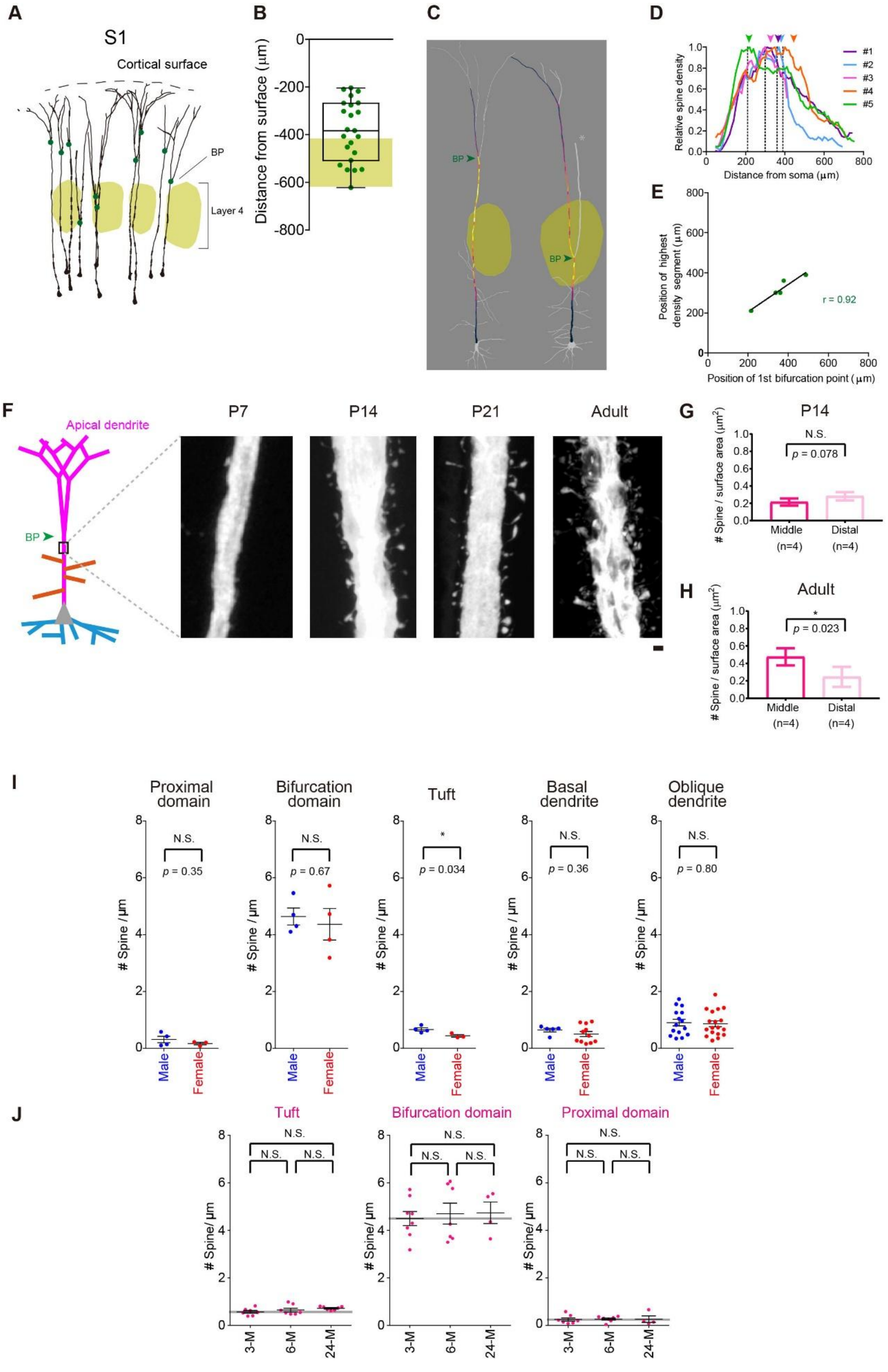
A
S1
Cortical surface
BP
Layer 4
B
Distance from surface (μm)
C
BP
D
Relative spine density
Distance from soma (μm)
#1
#2
#3
#4
#5
E
Position of highest density segment (μm)
Position of 1st bifurcation point (μm)
r = 0.92
F
Apical dendrite
BP
P7
P14
P21
Adult
G
P14
N.S.
p = 0.078
# Spine / surface area (μm²)
Middle (n=4)
Distal (n=4)
H
Adult
*
p = 0.023
# Spine / surface area (μm²)
Middle (n=4)
Distal (n=4)
I
Proximal domain
N.S.
p = 0.35
Bifurcation domain
N.S.
p = 0.67
Tuft
*
p = 0.034
Basal dendrite
N.S.
p = 0.36
Oblique dendrite
N.S.
p = 0.80
# Spine /μm
Male
Female
J
Tuft
Bifurcation domain
Proximal domain
N.S.
Spine/ μm
3-M
6-M
24-M

Fig. S3. Spine density hotspot in L5 ET neurons.

(**A**) The first bifurcation points (BP, green) are indicated for reconstructed thick-tufted L5 neurons in the adult brain (Thy1-YFP-H, age P84). Somata and apical dendrites were reconstructed and shown in black. Ten example neurons are depicted. Barrel structures in L4 were identified by VGluT2 immunostaining and shown in yellow.
(**B**) Distribution plot for the position of the first bifurcation points (distance from pia surface). Mean ± S.D. (n = 25) are also shown. L4 is indicated in yellow. The distribution of the bifurcation point was consistent with the normal distribution (p > 0.1, Kolmogorov–Smirnov test).
(**C**) Spine density mapping of two representative L5 ET neurons. Barrel structures (L4) are shown in yellow. The spine density hotspot was found near the first bifurcation point.
(**D**) The spine density hotspot was plotted (dotted lines) according to the distance from the soma for 5 neurons. Position of the first bifurcation point was indicated by arrowhead with corresponding color to each neuron.
(**E**) Correlation between the position of the first bifurcation point and the location of the hotspot (highest density segment determined every 10 µm). There was a correlation between the two parameters ($R^2 = 0.92$, $p < 0.01$, n = 5 neurons).
(**F**) Airyscan super-resolution images of the hotspot domains on apical dendrites at P7, P14, P21, and adult. Scale bar, 1 µm.
(G) Average spine density per dendritic surface area (spine number / μm^2) at the middle (200-500 µm from soma) and distal (>500 µm from soma) compartments of apical dendrite of S1 L5 ET neurons in P14 mice. Data are from **Fig. 3B-C**.
(H) Average spine density per dendritic surface area (spine number / μm^2) at the middle (200-500 µm) and distal (>500 µm) compartments of apical dendrites in S1 L5ET neurons in adult mice. Data are from **Fig. 3B-C**. Spine density in the middle compartment is significantly higher than in the distal part only in the adult. *$p < 0.01$, N.S., not significant (Student's t-test).
(**I**) Comparison of spine density between male and female at P63-84. *$p < 0.05$, N.S., not significant (Student's t-test).
(**J**) Spine density in apical dendrites in 3, 6, and 24-month-old animals. There is no reduction in spine density after 3 months. N.S., not significant (Sidak's multiple comparison test).

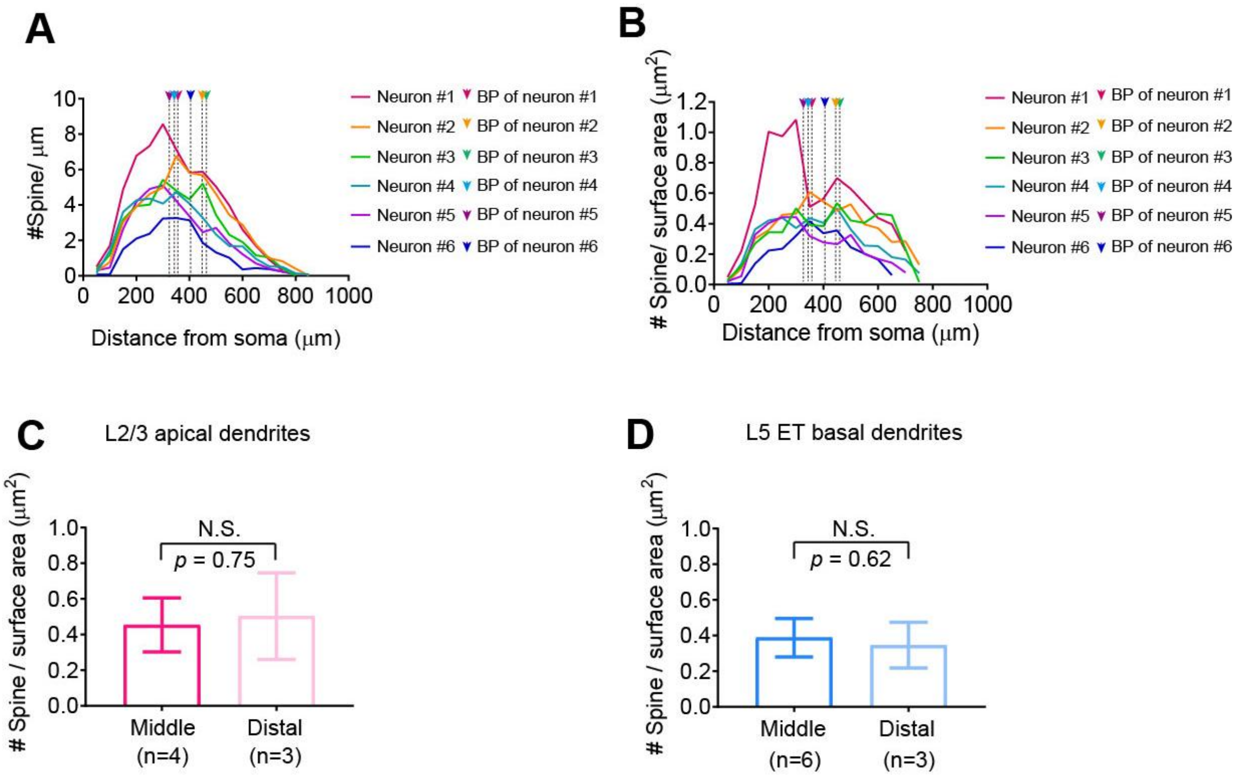

Fig. S4. Spine density in individual L5ET neurons in S1.

(**A**) Spine density per dendritic length quantified every 50 μm. Data are from 6 dendrites each (each from 3 mice). Neurons shown in Fig. 2 are shown.

(**B**) Spine density per dendritic surface area quantified every 50 μm.

(C) Average spine density per dendritic surface area (spine number / μm^2) at the middle (100-200 μm) and distal (>200 μm) compartments of the apical dendrite in S1 L2/3 neurons from adult mice. Data are mean ± SD. Data are from **Fig. 2G-J**.

(D) Average spine density per dendritic surface area (spine number / μm^2) at the middle (50-100 μm) and distal (100-150 μm) compartments of the basal dendrites in S1 L5ET neurons in adult mice. Data are mean ± SD. Data are from **Fig. 3D**.

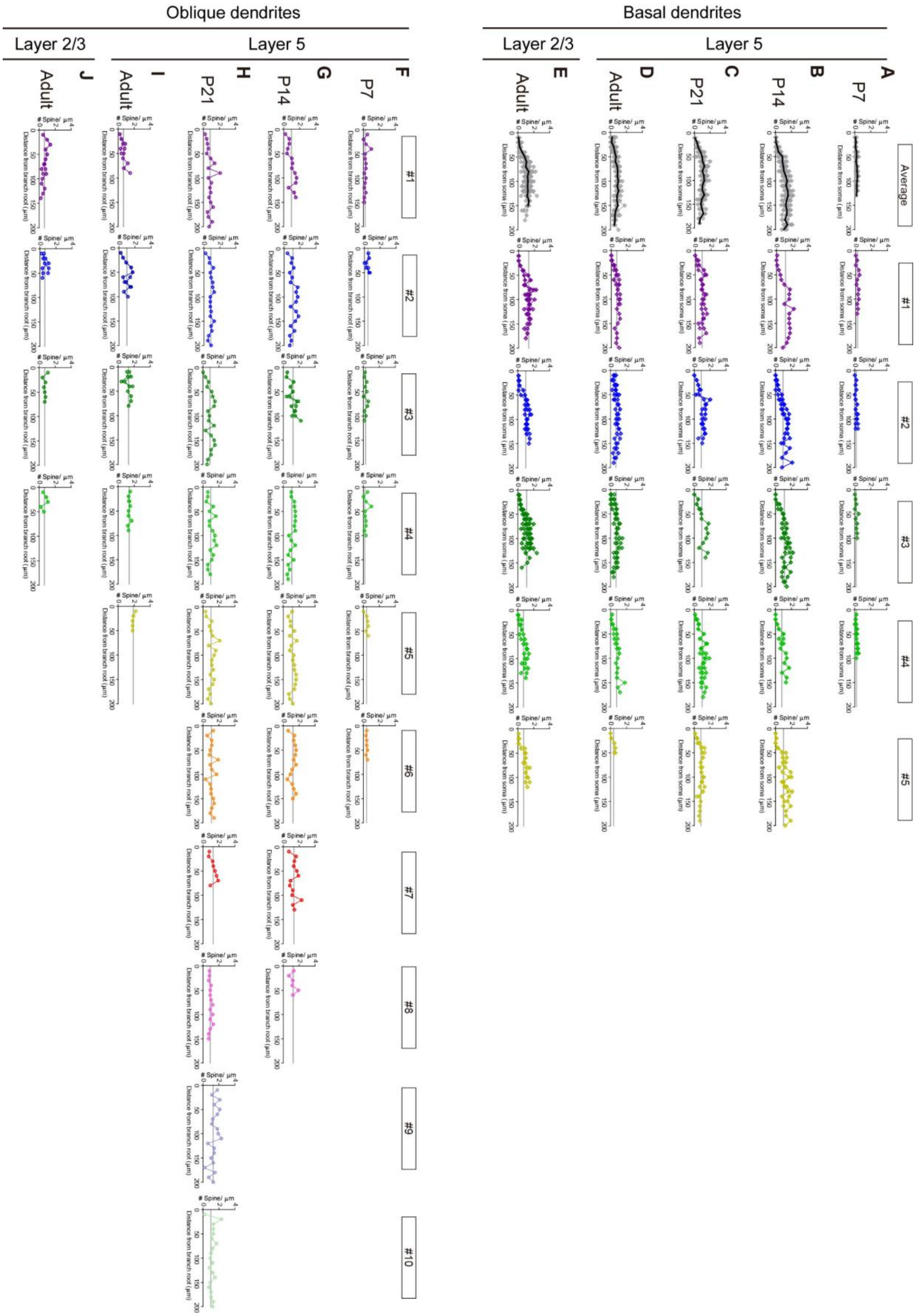

Oblique dendrites
Basal dendrites
Layer 2/3
Layer 5
Layer 2/3
Layer 5
A P7
B P14
C P21
D Adult
E Adult
F P7
G P14
H P21
I Adult
J Adult
Average
#1
#2
#3
#4
#5
#6
#7
#8
#9
#10
Spine/ µm
Distance from soma (µm)
Distance from branch root (µm)

Fig. S5. Spine density in individual dendrites.

(**A-E**) Representative sibling branches of basal dendrites in wild-type L5 neurons at P7 (**A**), P14 (**B**), P21 (**C**), adult (**D**), and adult L2/3 neurons (**E**). Gray horizontal lines indicate average spine density of the dendrite.
(**F-J**) Representative sibling branches of oblique dendrites in wild-type L5 neuron at P7 (**F**), P14 (**G**), P21 (**H**), adult (**I**), and adult L2/3 neuron (**J**). Gray horizontal lines indicate average spine density of the dendrite.

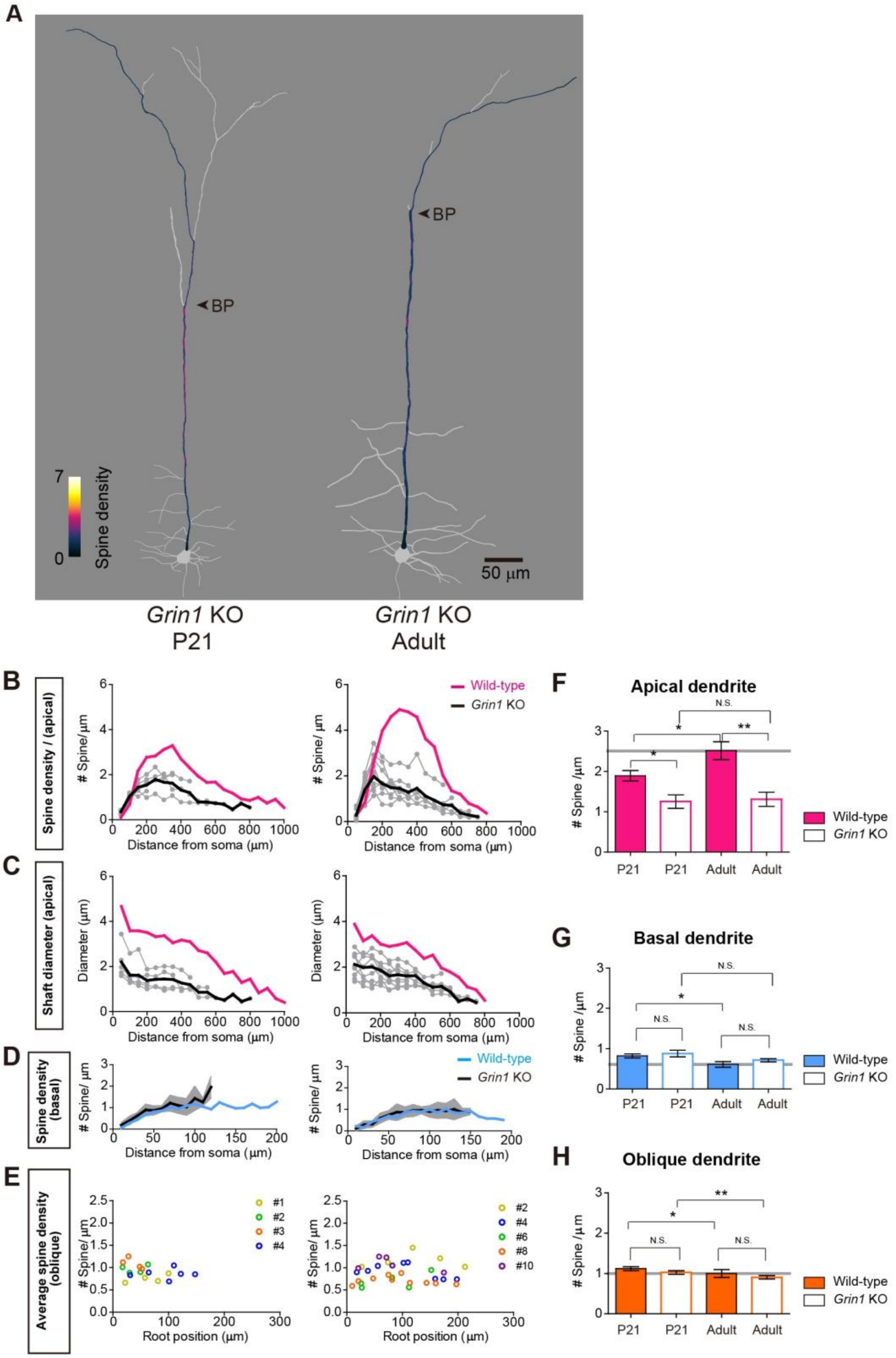
A
BP
BP
7
0
Spine density
50 μm
Grin1 KO
P21
Grin1 KO
Adult
B
Spine density / (apical)
Spine/μm
Distance from soma (μm)
Wild-type
Grin1 KO
C
Shaft diameter (apical)
Diameter (μm)
Distance from soma (μm)
D
Spine density (basal)
Spine/ μm
Distance from soma (μm)
Wild-type
Grin1 KO
E
Average spine density (oblique)
Spine/ μm
Root position (μm)
#1
#2
#3
#4
#2
#4
#6
#8
#10
F
Apical dendrite
Spine /μm
N.S.
*
**
*
Wild-type
Grin1 KO
P21
P21
Adult
Adult
G
Basal dendrite
Spine /μm
N.S.
*
N.S.
N.S.
Wild-type
Grin1 KO
P21
P21
Adult
Adult
H
Oblique dendrite
Spine /μm
**
*
N.S.
N.S.
Wild-type
Grin1 KO
P21
P21
Adult
Adult

Fig. S6. Single-cell *Grin1* KO.

(**A**) Spine density maps for Grin1 KO L5 ET neurons at P21 and adult (P77). *In utero* electroporation of the Cre plasmid was used to knockout *Grin1* in *Grin1*$^{fl/fl}$ mice. Representative reconstructions are shown.
(**B**) Spine density per dendritic length (spine number /µm). Red lines indicate wild type neurons (mean, shown in **Fig. 3B**). Thick black lines indicate *Grin1* KO neurons (mean). Gray lines indicate individual neurons. Data are plotted every 50 µm segment. Data are from 4 (P21) and 9 (adult) dendrites.
(**C**) Diameter of dendritic shafts at corresponding locations shown in (**B**).
(**D**) Spine density per length in basal dendrites (spine number / µm). Data are mean (thick lines) ± S.D. (shaded). Data are from 6 (P21) and 9 (adult) dendrites. See also **Fig. S5** for individual basal dendrite from one representative neuron.
(**E**) Spine density per length in oblique dendrites (spine number / µm). Root positions and average spine density in oblique dendrites were determined for multiple neurons. Each color indicates sibling oblique dendrites from the same neuron. Data are from 4 (P21) and 5 (adult) neurons. See also **Fig. S4** for individual oblique dendrite from one representative neuron.
(**F-H**) Average spine density (spine number / µm) in apical (**F**), basal (**G**), and oblique (**H**) dendrites in wild-type and *Grin1* knock-out neurons at P21 and adult. *P<0.05, **P<0.01, N.S., not significant (Mann-Whitney U test). Data are mean ± S.E.M. See **Table S1** for sample size. Scale bar, 50 µm.

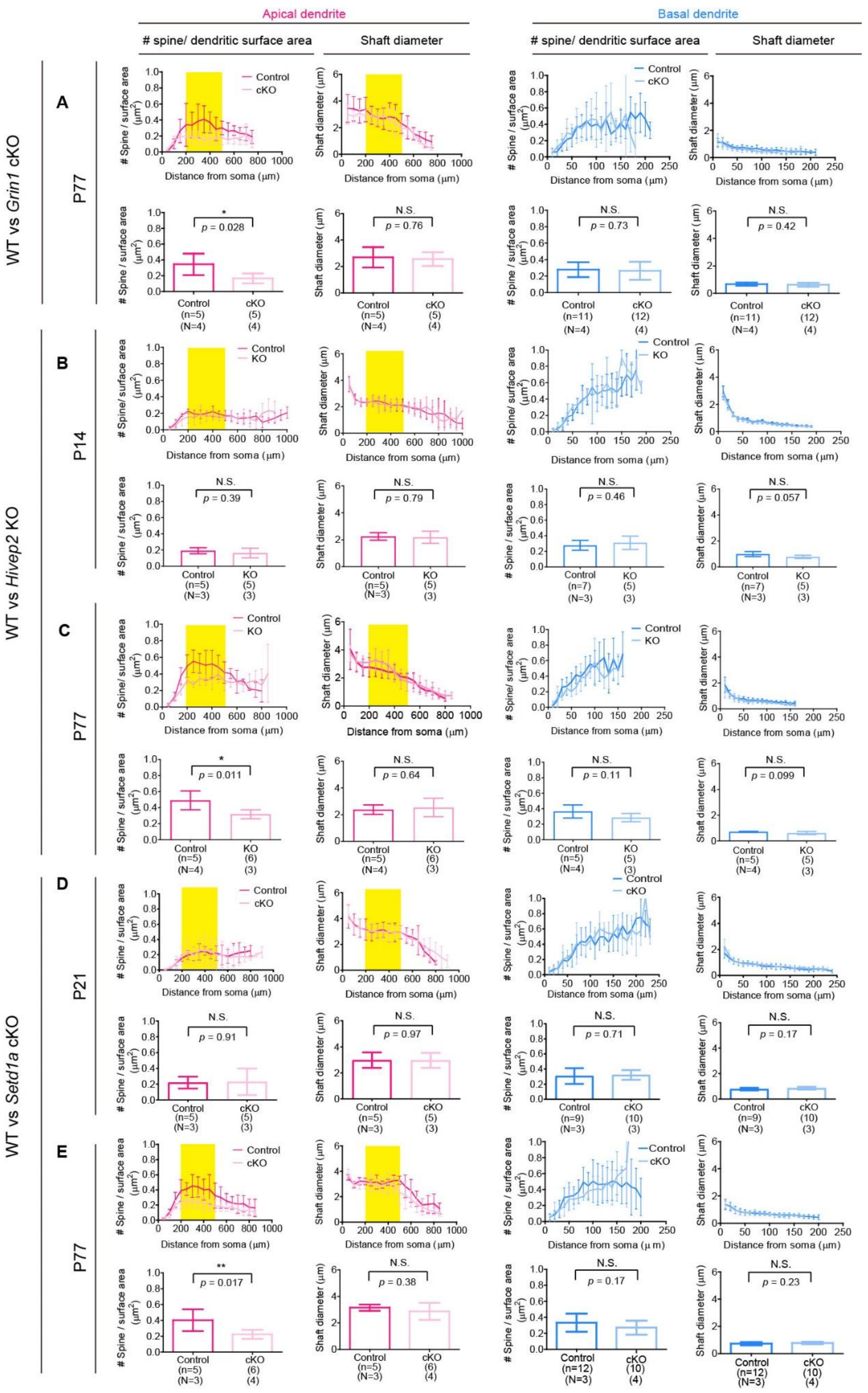

Apical dendrite
Basal dendrite
spine/ dendritic surface area
Shaft diameter
WT vs Grin1 cKO
WT vs Hivep2 KO
WT vs Setd1a cKO
A
B
C
D
E
P77
P14
P21
Control
cKO
KO
Spine / surface area (μm²)
Shaft diameter (μm)
Distance from soma (μm)
*
p = 0.028
N.S.
p = 0.76
p = 0.73
p = 0.42
p = 0.39
p = 0.79
p = 0.46
p = 0.057
p = 0.011
p = 0.64
p = 0.11
p = 0.099
p = 0.91
p = 0.97
p = 0.71
p = 0.17
**
p = 0.017
p = 0.38
p = 0.23

Fig. S7. Spine density per surface area and dendritic shaft diameter in mutants.

Spine density per dendritic surface area and shaft diameter in *Grin1* cKO (**A**), *Hivep2* KO (**B-C**) and *Setd1a* cKO (**D-E**). Top panels show spine density per dendritic surface area (spine number / μm^2) and shaft diameter along dendrites. Bottom panels show the average of spine density at the hotspot (shaded in yellow, 200-500 µm from soma) and basal dendrites. n, number of dendrites. N, number of mice. Data are mean ± S.D. *$p<0.05$, **$p<0.01$, N.S., not significant (Student's t-test).

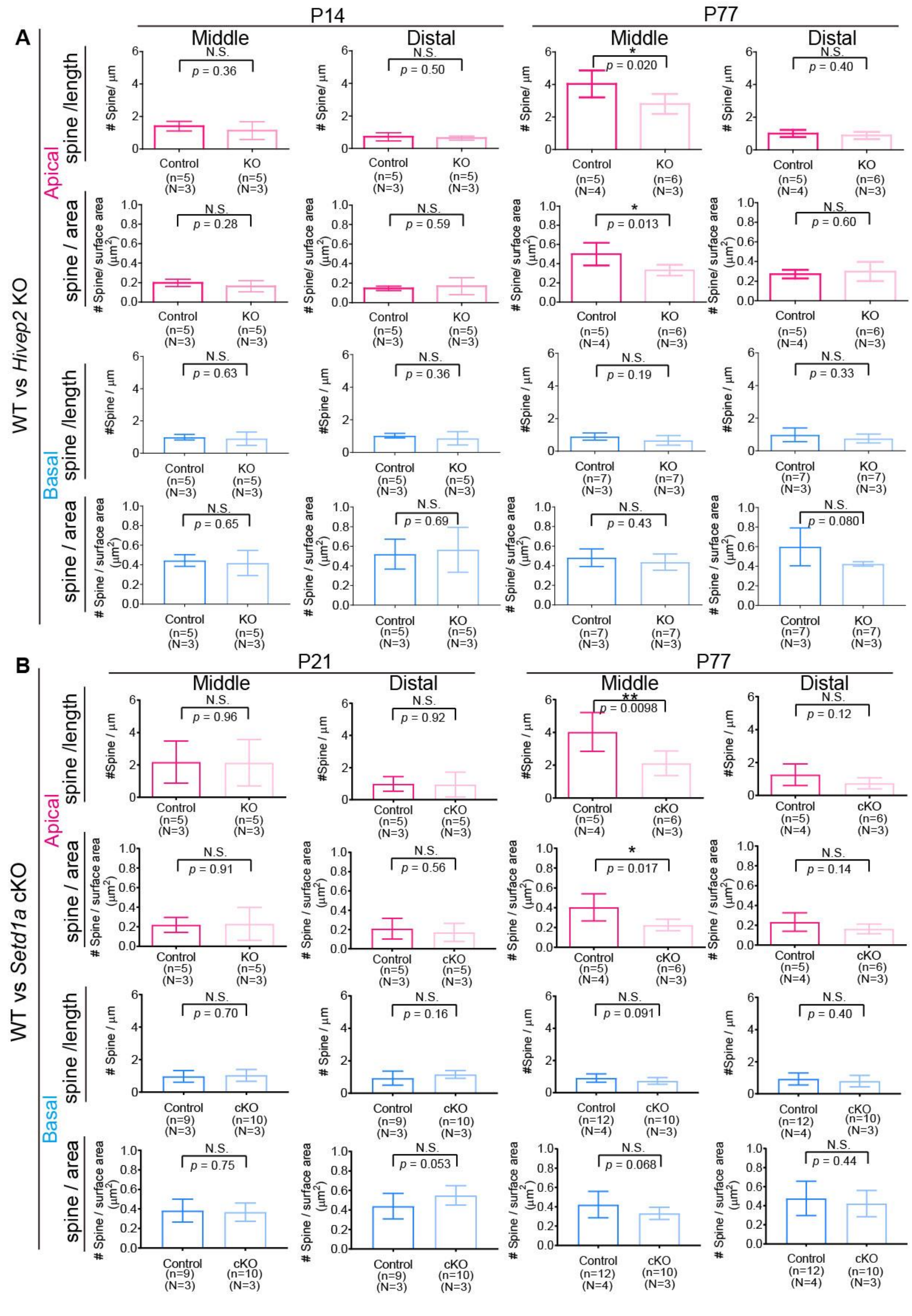

Fig. S8. Dendritic compartment-specific changes in spine density in mutants.

Spine density per length and surface area were analyzed at different dendritic compartments in apical and basal dendrites in *Hivep2* KO (**A**) and *Setd1a* cKO (**B**). The middle compartment is 200-500 μm for apical and 50-100 μm for basal dendrites; the distal compartment is >500 μm for

apical and 100-150 μm for basal dendrites. n, number of dendrites. N, number of mice. Data are mean ± S.D. *$p < 0.05$, (Student's t-test). See also Fig. 7. Samples are from **Fig. 7 and 8**.

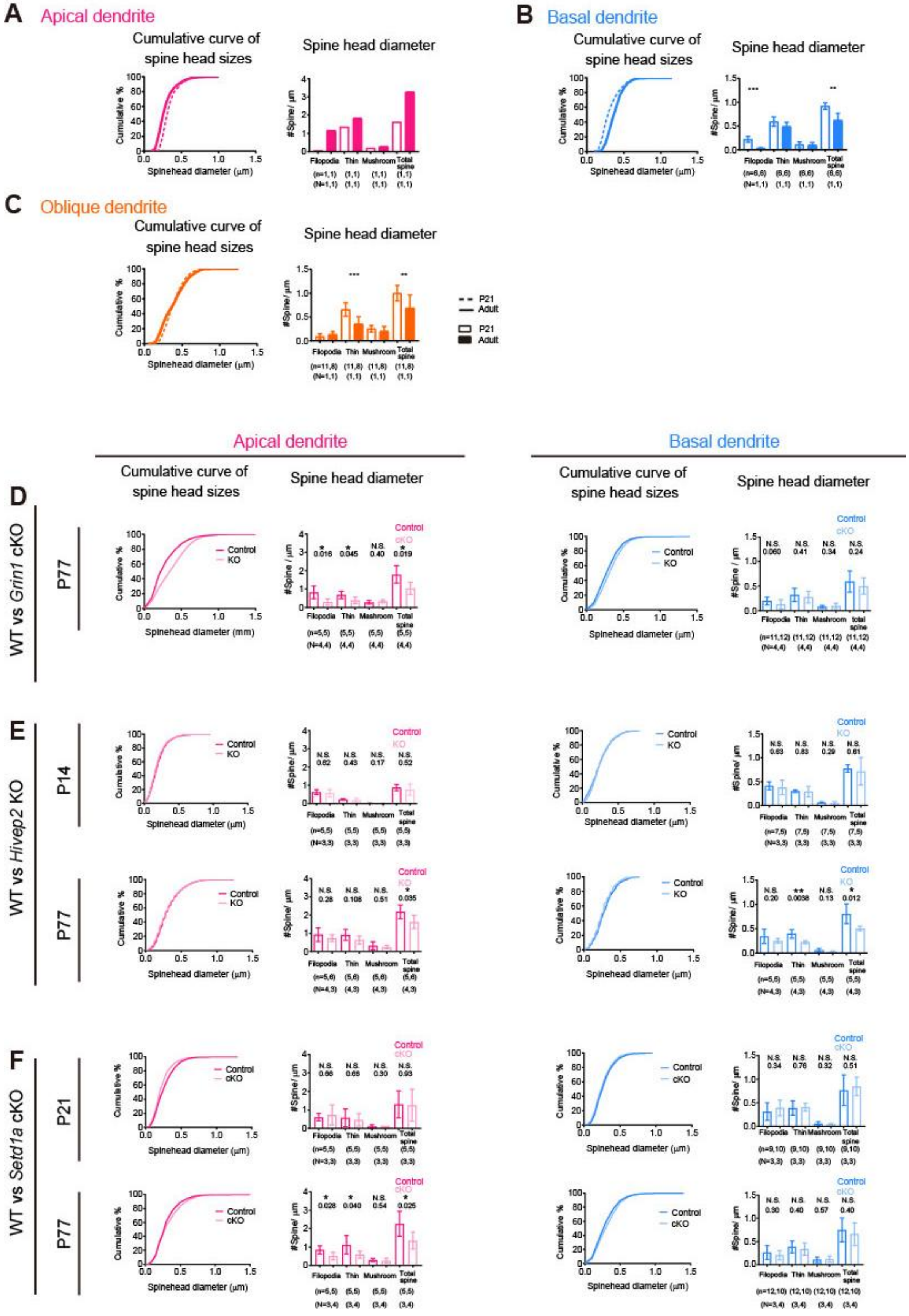
A
Apical dendrite
Cumulative curve of spine head sizes
Spine head diameter
B
Basal dendrite
Cumulative curve of spine head sizes
Spine head diameter
C
Oblique dendrite
Cumulative curve of spine head sizes
Spine head diameter
P21
Adult
P21
Adult
Cumulative %
Spinehead diameter (μm)
#Spine / μm
Filopodia
Thin
Mushroom
Total spine
Apical dendrite
Basal dendrite
Cumulative curve of spine head sizes
Spine head diameter
D
WT vs Grin1 cKO
P77
Control
KO
cKO
E
WT vs Hivep2 KO
P14
P77
F
WT vs Setd1a cKO
P21
P77
N.S.

Fig. S9. Spine size distribution in mutants.

(**A-C**) Cumulative curve of spine head sizes (diameter) for P21 and adult L5 ET neurons (left). Solid lines in cumulative curves indicate adult neurons and dotted lines indicate P21 neurons. Density of filopodia, thin spines, and mushroom spines in P21 and adult neurons (right).
(**D-F**) Cumulative curve of spine head diameter (left). Density of filopodia, thin spines, and mushroom spines (right). *Grin1* cKO (D) *Hivep2* KO (E) and *Setd1a* cKO (F) were analyzed. $*p < 0.05$, $**p < 0.01$, $***p < 0.001$, N.S., not significant (Student's t test). Data are mean ± S.D. Filopodia, thin spines, mushroom spines were defined based on the spine head diameter. Filopodia, <0.25µm; thin, 0.25-0.5 µm; mushroom, >0.5 µm. n, number of dendrites. N, number of animals.

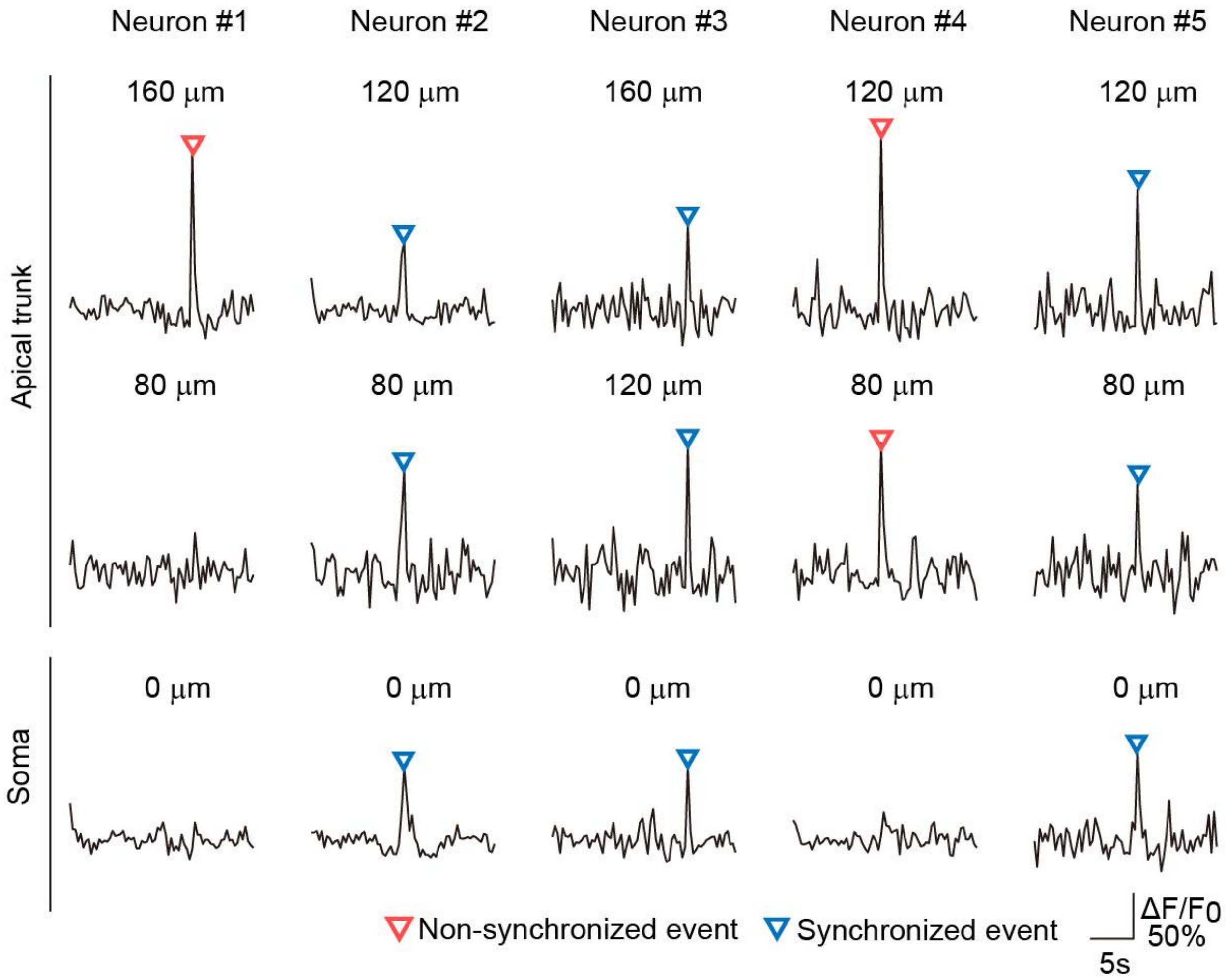

Fig. S10. ***In vivo*** **Ca^{2+} imaging of somata and apical dendrites in L5 ET neurons in awake mice**

Representative jGCaMP8m signals (ΔF/F) at the soma and in each part of the dendrites. *In vivo* Ca^{2+} imaging of the L5 ET neurons in awake mice (2-month old) was performed. L5 ET-specific AAV-Cre (mscRE4-Cre) was used in combination with a Cre-dependent AAV vector to sparsely label L5 ET neurons with jGCaMP8m and CyRFP1 (filler). Using a fast piezo z-axis drive, we imaged a 160 μm-thick volume (5 planes / volume) spanning the soma and dendrites. The distances from the soma are indicated in the panels. Ca^{2+} "events" were defined as follows: i) Peak ΔF/F > 50% and ΔF/F > baseline (5 frames) + 3SD, or ii) Peak ΔF/F > 50% and the peaks are detected in multiple ROIs. Pink arrowheads indicate non-synchronized events (i.e., local dendritic spikes) only seen in the dendrite. Blue arrowheads indicate synchronized events between the soma and the dendrites. The synchronized events often (but not always) accompanied the somatic action potentials (e.g., neuron#2, 3, 5). In contrast, non-synchronized dendritic spikes did not accompany somatic action potentials (e.g., neuron#1).

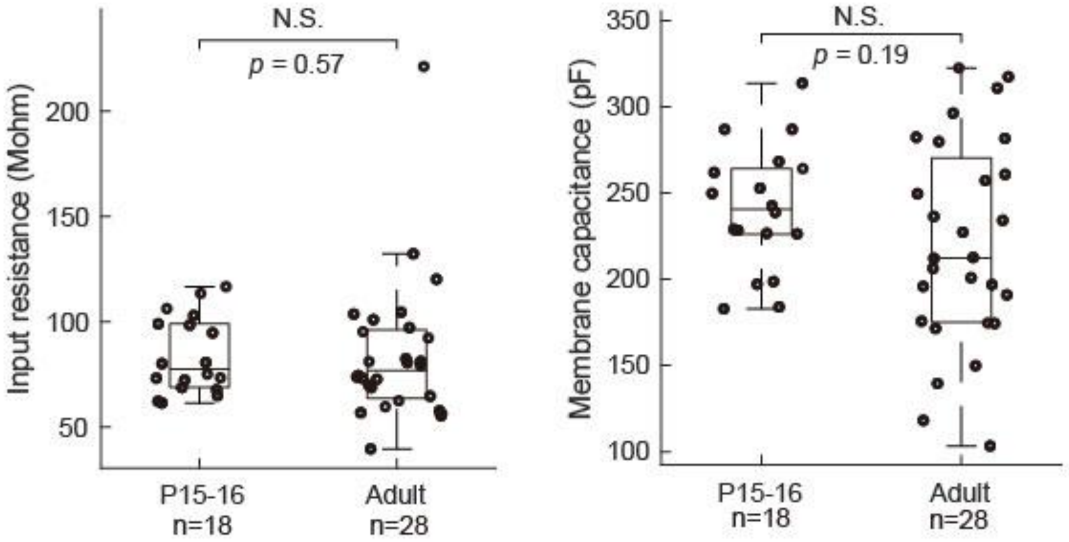

Fig. S11. Electrophysiological recording of L5 ET neurons in acute brain slices.

Input resistance and membrane capacitance of Thy1-YFP-H^+ L5 ET neurons at P15-16 and adult (P64-82). Samples are the same as in **Fig. 9E-I**. Box plots indicate median ± IQR. N.S., non-significant (Wilcoxon rank sum test).

Table S1. The number of dendrites, neurons, and animals analyzed in each experiment (separate Excel file).

Table S2. Numerical source data (separate Excel file).

Movie S1. Visualization of the entire length of an apical dendrite of a L5 ET neuron. Volume rendering of super-resolution images is shown. See also **Fig. S2**.
